# Mechanism of Polyester Hydrolysis by Marine Bacterium PE‑H Enzyme: an Atomistic and Thermodynamic Characterization

**DOI:** 10.1021/acs.jcim.5c02314

**Published:** 2026-02-12

**Authors:** Samah Nassir, Pedro Paiva, Rui P. P. Neves, Pedro A. Fernandes, Achraf El Allali, Maria J. Ramos

**Affiliations:** † Associated Laboratory for Green Chemistry (LAQV)/Network of Chemistry and Technology (REQUIMTE), Departamento de Química e Bioquímica, Faculdade de Ciências Universidade Do Porto, Rua Do Campo Alegre, s/n, Porto 4169-007, Portugal; ‡ Bioinformatics Laboratory, College of Computing, 479571 University Mohammed VI Polytechnic, Lot 660, Hay Moulay Rachid, Ben Guerir 43150, Morocco

## Abstract

Polyethylene terephthalate (PET) is a widely used plastic due to its durability and adaptability; however, its resistance to natural degradation has led to severe accumulation in the environment. Recently, a PET-degrading marine bacterium, *Pseudomonas aestusnigri*, was identified and proposed for possible use in sustainable plastic recycling, particularly the PE-H enzyme, which hydrolyses PET with MHET as the main hydrolysis product. In this work, we investigate the reaction mechanism of PE-H through umbrella sampling hybrid quantum mechanics/molecular mechanics molecular dynamics simulations at the PBE/AMBER level. Our results show a two-stage reaction pathway: acylation and deacylation, both of which proceed stepwise via tetrahedral intermediate formation. We identified deacylation as the rate-limiting step with a free energy barrier of approximately 10.6 kcal·mol^–1^, which is relatively lower than the barrier of other PET hydrolyses. Our analysis suggests that structural features promoting oxyanion hole formation or enhancing substrate accommodation contribute to lower free energy barrier and promote catalysis. We highlight the role of the S171, H249 and D217 triad responsible for the catalysis of proton transfer and nucleophilic attack reactions, and of F98 and M172 responsible for the formation of the oxyanion hole contributing to the stabilization of tetrahedral intermediates formed along the path. These findings provide mechanistic insights into PE-H catalysis and suggest structural factors that could be extended to other enzymes, providing a basis for future studies to understand enzymatic plastic degradation.

## Graphical abstract

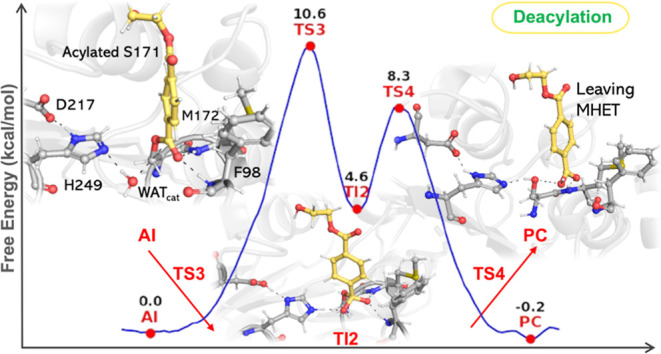

## 1 Introduction

Modern society relies heavily on plastics because they are durable, resilient, affordable and widely available, leading to a serious and growing risk of waste accumulation both on land and marine environments.^1^ According to the United Nations Environment Programme (UNEP), the annual production of plastics exceeded 365 million tons in 2020,^1^ almost half of which was destined for single-use applications.^2^ Single-use plastics, such as those found in packaging, disposable cutlery, tableware and plastic bottles, have become a particular challenge.^2^ Packaging alone accounts for around 40% of total plastic consumption,^3^ disposable cutlery and tableware contribute to the 1 million plastic utensils used every minute worldwide.^2^ Plastic bottles are another major problem: over 1 million bottles are purchased worldwide every minute, and only 9% of these bottles are recycled. Polyethylene terephthalate (PET) is one of the most well-known single-use plastics. The production of PET is generally based on either esterification or transesterification processes, each of which is followed by polycondensation (Figure 1a).^4^ During esterification, terephthalic acid (TPA) reacts with ethylene glycol (EG), while dimethyl terephthalate (DMT) is used instead of terephthalic acid (TPA) during transesterification. The resulting intermediates are then condensed to form the long-chain PET polymer. PET is a semicrystalline thermoplastic polyester whose ester groups exhibit more resistance to biodegradation compared to other polymers.^5^ Together with polyethylene (PE), polypropylene (PP) and polystyrene (PS), it is an important component of plastic waste in the environment.^5,6^ The increasing dominance of this material poses a major threat to ecosystems, biodiversity and human health. Current strategies for managing PET waste include landfilling, incineration, and recycling. However, landfilling and incineration can contribute to groundwater contamination and CO_2_ emissions, while recycling often compromises material quality and can prove economically impractical.^5^ With almost 70% of plastic waste either landfilled or incinerated, 9% recycled and the rest ending up in the environment, there is an urgent need for more efficient solutions.^7^ Enzymatic degradation of plastics has emerged then as a promising, eco-friendly and cost-effective alternative offering a potential path toward better management of plastic waste.

**Figure 1:**
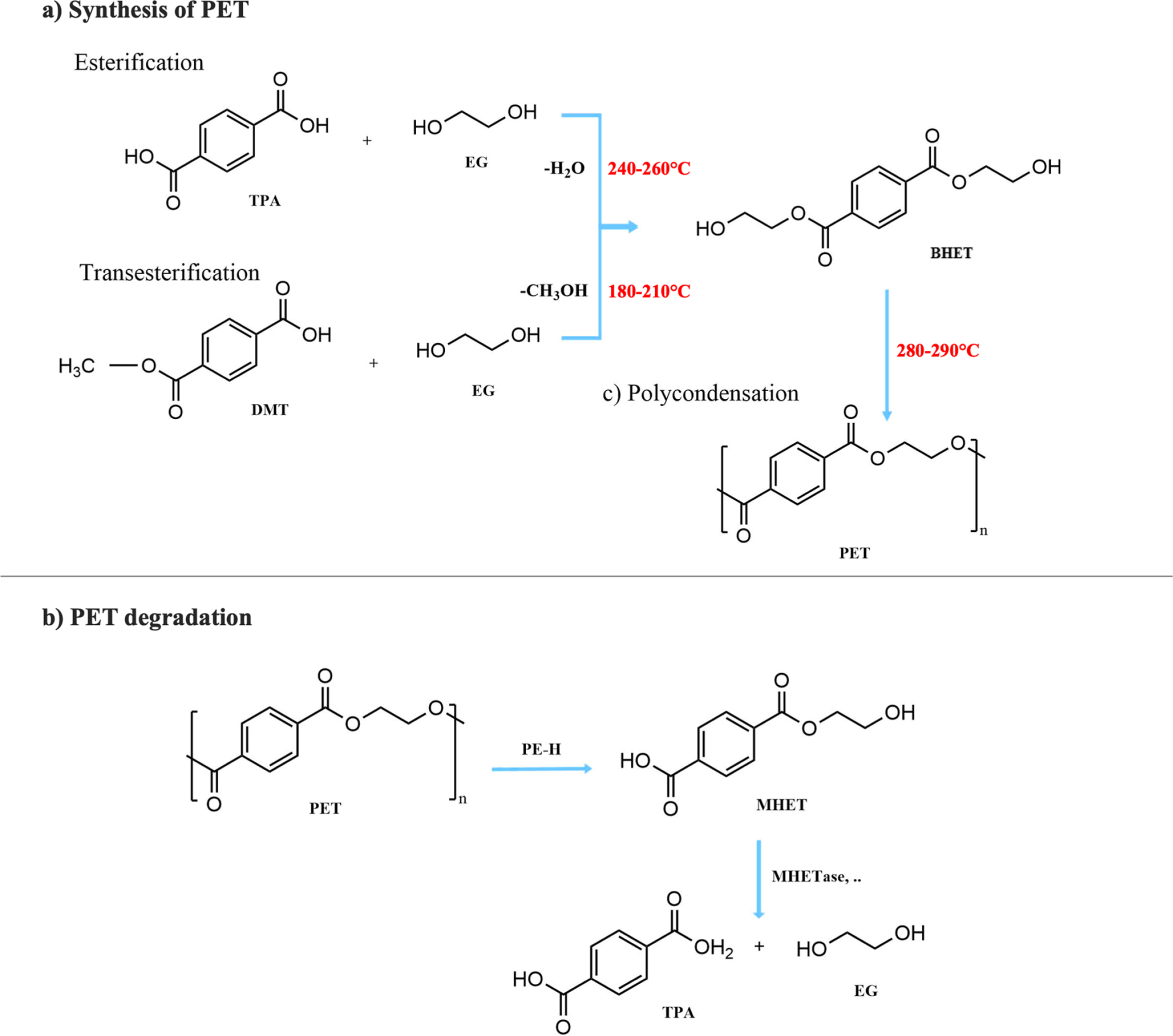
(a) Polyethylene terephthalate (PET) is synthesized via two pathways. Esterification: terephthalic acid (TPA) reacts with ethylene glycol (EG) at high temperature to form bis­(2-hydroxyethyl) terephthalate (BHET) with water elimination. Transesterification: dimethyl terephthalate (DMT) reacts with EG at low temperature to form BHET with methanol elimination. Both pathways produce BHET as an intermediate, which can be polymerized at high temperatures to produce PET. (b) PET can be degraded into mono-(2-hydroxyethyl) terephthalate (MHET) by PE-H. MHET can later be hydrolyzed to terephthalic acid (TPA) and EG by MHETase or similar enzymes.^4^

Recent findings highlight the significant potential of enzymatic degradation, especially of polyester hydrolases, enzymes that catalyze the degradation of ester bonds in polyesters, as an important focus in the search for efficient biodegradation solutions.^8^ Among these enzymes, polyester hydrolase (PE-H), which was discovered in 2020 by Alexander Bollinger and co-workers,^9^ has attracted considerable attention due to its unique enzymatic properties and proven effectiveness in the degradation of PET. PE-H was identified in the marine bacterium *Pseudomonas aestusnigri* VGXO14 as a member of the PET hydrolase type IIa family, and it is characterized by its ability to hydrolyze PET at moderate temperatures (≈30 °C).^9^ The enzyme has a high structural identity and about 48–51% sequence similarity with other PET-hydrolytic enzymes. PE-H primarily converts amorphous PET to mono­(2-hydroxyethyl) terephthalate (MHET) (Figure 1b).^9^ For crystalline PET, the efficacy of PE-H is lower than that of other enzymes such as *Thermobifida fusca* cutinase (TfCut2) and PET hydrolase from *Ideonella sakaiensis* (PETase), which showed higher activity toward crystalline PET substrates.^10^ However, although the activity of PE-H was significantly lower, and no hydrolysis products were detectable, Bollinger et al. have shown that PE-H acquires the ability to effectively degrade both amorphous and crystalline PET substrates when the single mutation Y250S is introduced. These results highlight the potential of PE-H, with its ability to accommodate various large substrates, as a model for studying the mechanisms of PET degradation, as they provide a clear pathway for the development of variants with improved substrate specificity and catalytic efficiency.

### 1.1 Structural Insights into PE-H

The X-ray crystal structure of PE-H was resolved at the high resolution of 1.09 Å (PDB ID: 6SBN).^9^ The structure shows a typical α/β-hydrolase fold, which is known to be a characteristic feature of many enzymes associated with hydrolysis reactions. It reveals a central twisted β-sheet consisting of nine β-strands adjacent to seven α-helices (Figure 2a), which form a stable backbone essential for enzymatic activity. The active site of PE-H is crucial for its hydrolytic function and contains a catalytic triad of serine (S171), histidine (H249), and aspartate (D217), which cooperate to facilitate the nucleophilic attack on the ester bond of PET. The oxyanion hole formed by the backbone NH groups of M172 and F98 is proposed to stabilize the tetrahedral intermediate during the hydrolysis reaction and increase the enzyme’s catalytic efficiency.^9^ In addition, the structure has two disulfide bonds connecting the cysteine residues C214–C251 and C285–C302, another common structural motif in PET-degrading enzymes.^11^ The two disulfide bridges provide stability to the structure of the enzyme and maintain the integrity of the active site. The active site cleft of PE-H is significantly wider and shallower compared to other PETases, and that presumably improves its ability to accommodate different substrate conformations.

**Figure 2:**
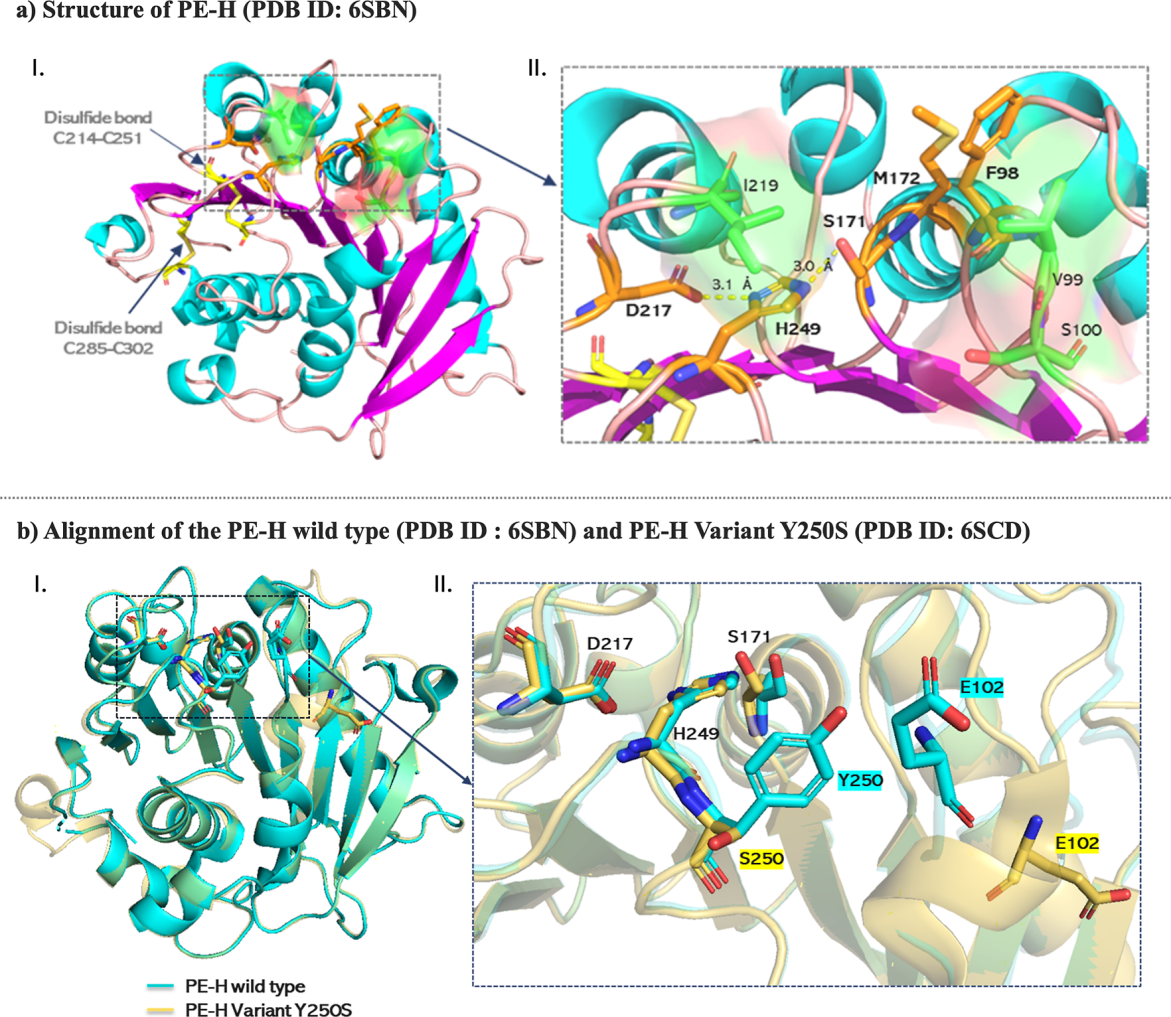
(a) I. The crystal structure of PE-H is represented as a cartoon, with β-strands in magenta, α-helices in cyan, and loops in light pink. The residues that form the catalytic triad and oxyanion hole are represented as orange sticks. The cysteine residues that establish disulfide bonds are shown as yellow sticks. The residues involved in the arrangement of the loop connecting the β3-α2 motif, which influences substrate accessibility, are shown as green sticks, with their spatial arrangement highlighted as a surface. II. Close up view of the residues involved in the arrangement of the loop connecting the β3-α2 motif, and residues that form the catalytic triad and oxyanion hole, with the critical interaction distances given in Å. (b) I. Superposition of the overall structures of the wild-type (cyan) and the Y250S variant (yellow), with key catalytic and substrate-binding residues shown as sticks, demonstrating the global structural similarity. II. Close-up view of the aligned active sites, with key residues labeled. The Y250S mutation caused a new rearrangement and created space that resulted in a more accessible active site and eliminated the polar contact between the hydroxyl group of Y250 and the backbone amine of E102.

Other key amino acid residues were identified as crucial for the activity of the enzyme. The residues valine (V99), serine (S100), and isoleucine (I219) are particularly important as they are involved in the arrangement of the loop connecting the β3-α2 motif and influence access to the substrate (Figure 2a). In addition, the introduction of mutations such as Y250S significantly increases the activity of the enzyme toward both amorphous and crystalline PET. The X-ray crystal structure of the Y250S variant solved at 1.35 Å (PDB ID: 6SCD) has shown a new arrangement of the active site that allows better substrate accommodation. The structural comparison between the wild-type PE-H and the Y250S variant shows that the latter exhibits a rearrangement of the active site conformation favored by the prevention of polar contact between the hydroxyl group of Y250 and the backbone amine of E102, resulting in an increase in the active site cavity volume from 153 Å^3^ in the wild-type to 362 Å^3^ in the variant (Figure 2b). These insights into the structural features, including the catalytic triad, oxyanion hole, disulfide bonds, and overall fold of PE-H, underscore its potential for further research and application in biodegradation and highlight the importance of understanding the molecular basis of its activity for biotechnological applications.

### 1.2 Suggested Catalytic Mechanism of PE-H

Bollinger et al. performed molecular docking with the substrates MHET, Bis­(2-hydroxyethyl) terephthalate (BHET), and a previously described PET tetramer^11^ to provide insight into the protein-substrate interactions between PE-H and PET. Curiously, the wild-type PE-H did not bind the investigated substrates in the active site, but the substrate molecules accumulated in a nearby groove. The two unique binding poses found for the PE-H Y250S variant suggest a catalytic mechanism for the polyester hydrolase PE-H that begins with substrate binding in an adjacent groove near the active site, which could facilitate the hydrolysis of PET by anchoring the polymer chain. The adjacent groove aids in substrate binding and allows for a processive mechanism in which one unit of the polymer can bind to the groove while another unit bridges the distance to the catalytic site, thereby enhancing the ability of the enzyme to cleave the polymer chain. This proposed binding mechanism aligns with the observations of other PET hydrolases such as PETase and Cutinase, which have similar substrate binding modes that stabilize different units of a polymeric substrate during degradation.^11^ The similarities of these strategies suggest that anchoring mechanisms may be a common feature among PET-degrading enzymes. Since there is currently no detailed mechanism for PE-H, we propose a mechanism based on the demonstrated similarity with PETase.^12^ The proposed mechanism suggests that during acylation the catalytic serine residue (S171) acts as a nucleophile attacking the carbonyl carbon of the ester bond in the substrate. The catalytic H249 plays a crucial role by deprotonating the nucleophilic S171, while D217 stabilizes the positive charge of H249 during the reaction. The nucleophilic attack leads to the formation of a short-lived tetrahedral intermediate (TI) maintained by an oxyanion hole formed by hydrogen bonding of the backbone NH groups of M172 and F98 to its negatively charged carbonyl oxygen. The tetrahedral intermediate collapses, leading to the cleavage of the ester bond and the release of MHET. During deacylation, the mechanism involves a catalytic water molecule acting as a nucleophile attacking the carbonyl carbon of the acylated serine, leading to the breakdown of the latter, the consequent release of MHET and the regeneration of the enzyme’s active site (Figure 3).

**Figure 3:**
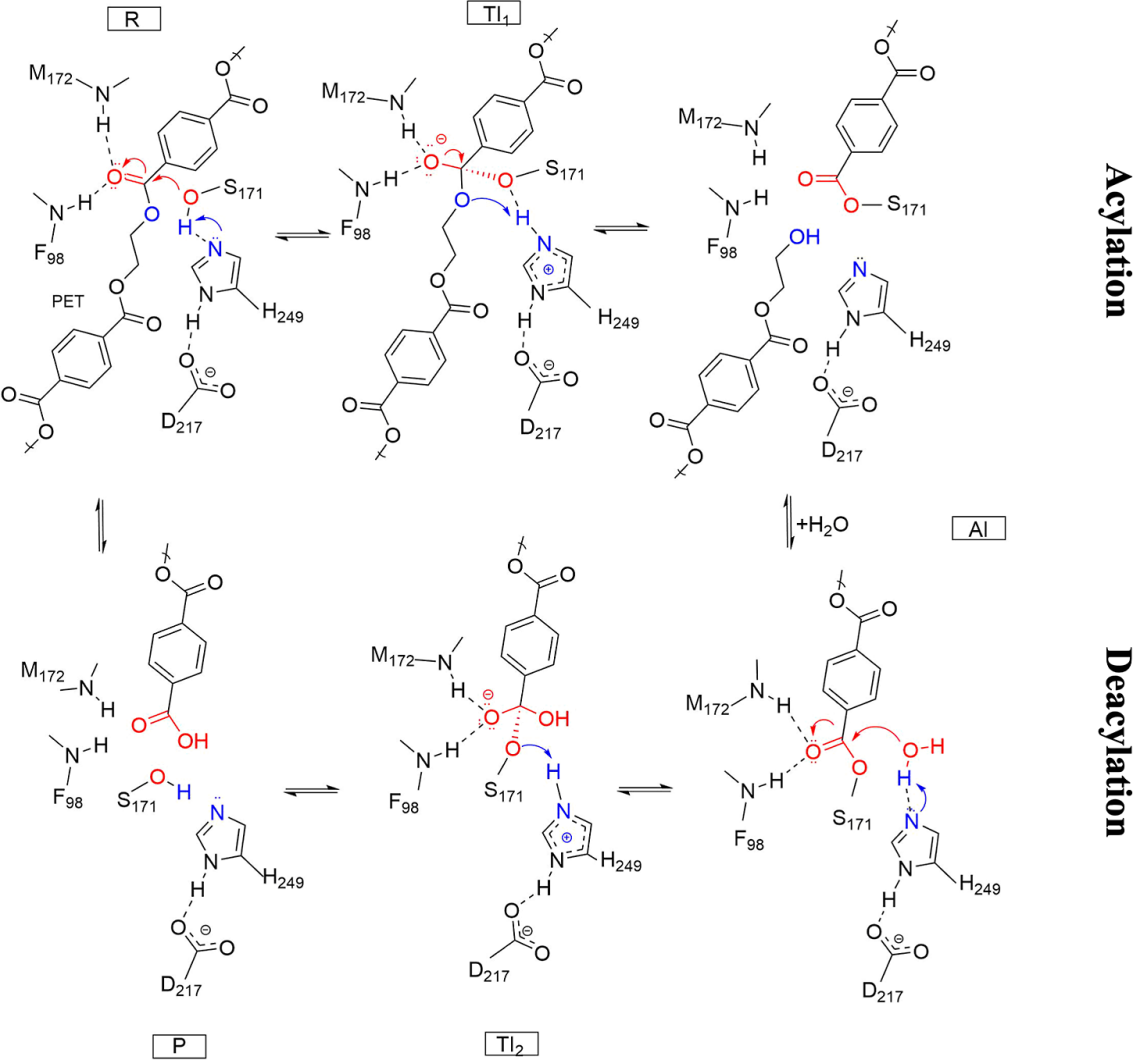
Proposed reaction mechanism of PE-H following the canonical serine hydrolase mechanism. The reaction proceeds via: (R) binding of PET to the active site of PE-H; (TI1) nucleophilic attack by the catalytic serine (S171) on the carbonyl carbon of the PET ester bond forming a tetrahedral oxyanion intermediate, which is stabilized by the oxyanion hole; (AI) collapse to an acyl-enzyme intermediate with simultaneous release of the first alcohol product; (TI2) hydrolysis of the acyl-enzyme complex via water activation by the histidine-aspartate pair, thereby regenerating the active site (P).

Building upon mechanistic insights into PETase and Cutinase,^12,13^ which proceed through a four-step pathway involving initial nucleophilic attack, formation of a tetrahedral intermediate, acylation to form an acyl-enzyme intermediate, and later hydrolysis (deacylation) to release the product, it is likely that PE-H follows a similar mechanism. However, variations in structure could affect the precise energetics and rate-limiting step, justifying forthcoming computational research to confirm this proposed mechanism. Computational studies, such as those presented by Boneta et al. employing M06-2X functional with the 6–31 + G­(d,p) basis set and Jerves et al. at the PBE level with DZVP-GTH basis and GTH pseudopotentials.,^12,13^ indicate that the acylation stage is generally the rate-limiting step (∼18–21 kcal·mol^–1^) and is similar across these enzymes, with the process proceeding in a stepwise manner. However, a study by Guo et al. using M06-2X/MM-MD,^14^ reveals that the deacylation stage is the slowest, with a free energy barrier of about 16.3 kcal·mol^–1^, making it the rate-limiting step under their conditions.

The present study focuses on the detailed analysis of the reaction mechanism of PE-H, involving mapping out the reaction pathway through a comprehensive QM/MM umbrella sampling method, at the density functional theory level (PBE), to identify key intermediates and transition states and calculate the associated free energy barriers. These findings will provide a detailed understanding of the catalytic efficiency of PE-H and could guide future modifications to improve the biodegradation of PET.

## 2 Methods

### 2.1 Model Preparation

The PE-H/substrate model was built from chain A of the crystal structure of the Y250S mutant PE-H (PDB ID: 6SCD, 1.35 Å resolution), as it has no missing residues and has a wider active site cleft. We used the mutagenesis tool in PyMOL software^15^ to revert the Y250S mutation to its original state. We also removed all water molecules and ligands as well as all duplicate atoms (S100-F130-M59-M172-S203-S235-F243-Y278-S291). While increasing the PET oligomer length can modulate the activation barriers through additional enzyme–substrate interactions, prior studies show that the reaction mechanism remains unchanged.^14,15^ Accordingly, and in line with previous mechanistic QM/MM studies,^13,14^ a PET dimer was chosen as the model substrate and PyMOL was used to build the PET dimer (i.e., the substrate). We started by modeling a PE-H/MHET complex based on the MHET present at the active site of the ICCG variant of the leaf branch compost Cutinase structure (PDB ID: 7VVE, 1.95 Å resolution),^16^ as the ester carbon of the ligand was close to residue S131A (3.1 Å). In addition, the structure retained the conserved terephthalate moiety of PET and exhibited 49% sequence similarity to the PE-H structure and an all-atom root-mean-square deviation (RMSD) of 1.34 Å. The complex of PE-H binding to a PET dimer was then obtained using the Genetic Optimization for Ligand Docking (GOLD) software, and ChemPLP to score the resulting PET dimer poses.^17,18^ We used MHET from the modeled PE-H/MHET complex as the reference template during the docking of the PET dimer, and all atoms within 6 Å of the reference ligand were considered to define the binding site of the PET dimer. Additionally, we set the ligand’s ring conformations to match the conformations of the template MHET. Initial unconstrained docking of the PET dimer was performed to assess whether promising catalytic poses were obtained, from which resulted four promising binding poses (Figure S1 in Supporting Information). We then constrained the distance between the ligand’s ester carbon and residue S171 of the catalytic triad to 1.5–3.2 Å using a harmonic spring constant of 5.0 score units·Å^–2^. In addition, the distances between the ligand’s carbonyl group (bearing the ester carbon) and the residues proposed to form the oxyanion hole, F98 and M172, were restricted to 1.5–2.9 Å and 1.5–3.2 Å, respectively, with the same spring constant. These restraints were applied to encourage sampling of poses with catalytic potential, yielding six poses favorable for catalysis (Figure S2 in Supporting Information). The final PET dimer geometry was chosen from the latter docking calculation because it was similar in both docking calculations and exhibited slightly better distances for catalysis. The Gaussian 09 software^19^ was used to optimize the PET dimer substrate’s geometry at the HF/6-31G­(d) level to calculate the RESP^20^ atomic point charges. Intramolecular bonds and the substrate’s Lennard-Jones parameters were parametrized using the generic Amber force field (GAFF2).^21^ The antechamber module of the Amber software suite^22^ was used to assist the parametrization of the PET dimer. To address p*K*_a_ values in protein complexes, structural problems including misplaced hydrogens, unbonded disulfide bridges, and missing atoms must frequently be fixed prior to p*K*_a_ estimation. In that regard, protonation states were assessed with the PROPKA^23^ program to predict the p*K*_a_ values of each residue in the complex at a physiological pH of 7.0 and were subsequently confirmed by visual inspection. Hydrogens initially generated by GOLD were removed, and the FF14SB^24^ force field in Xleap was used to correct protonation states and add missing atoms. Additionally, two disulfide bonds were defined between residues C214–C251 and residues C285–C302 using Xleap. According to PROPKA predictions, residues H225 (p*K*_a_ = 4.78), H273 (p*K*_a_ = 5.60), and H289 (p*K*_a_ = 5.20) were protonated at the ε-nitrogen, in line with the hydrogen bonding networks involving neighboring residues. In contrast, H249 (p*K*_a_ = 5.99) was protonated at the δ-nitrogen to serve as the catalytic base, enabling the deprotonation of the serine residue during the reaction mechanism (as represented in Figure 3). The total charge of the system was −2 and it was neutralized with two sodium ions. The complex was centered in a rectangular water box extended by 12 Å beyond any protein atom and solvated with TIP3P^25^ water molecules. The final system consisted of a total of 37,658 atoms. For the deacylation stage, the structural model was constructed based on the product of the last acylation stage, using the same parametrization as for the acylation stage to ensure the consistency of our computational approach. The acylated serine was parametrized according to the standard AMBER protocol for modified residues (parameters are provided in Supporting Information). The acylated serine was built and parametrized in its ACE-capped (N-terminus) and NME-capped (C-terminus) form, allowing a proper description of its chemical environment during parameter derivation. Parameters were generated using GAFF2 and subsequently integrated with the FF14SB protein force field for use in the MD simulations.

### 2.2 Molecular Dynamics Simulations

For the assembled molecular system, a five-step minimization protocol was performed to allow the system to adapt to the modeling procedure (details provided in the Supporting Information). The system’s geometry optimization was performed in GROMACS^26^ using the steepest descent algorithm. Electrostatic interactions were calculated using the Particle Mesh Ewald (PME) method^27^ beyond a cutoff distance of 10 Å, which was used for both Coulomb and van der Waals interactions. Periodic boundary conditions were applied in all directions. A heating (annealing) phase was performed at constant *NV* for 500 ps. The reference temperature from the experimental data of 303.15 K was chosen as the target temperature, in agreement with literature.^9^ The subsequent equilibration and production runs were performed in the *NPT* ensemble at 303.15 K and 1 bar, regulated by the canonical sampling velocity rescaling (CSVR) thermostat and a Berendsen barostat. The first MD run lasted 2 ns, with position restraints applied to the backbone amides of residues F98 and M172, reported to form an oxyanion hole with the oxo group of the substrate’s ester, and to the carbonyl group of the substrate involved in the oxyanion hole. Subsequently, a 300 ns and a 100 ns production run was performed without restraints for the initial composition of the acylation and deacylation steps, respectively. To evaluate the system stability, the RMSD and the root-mean-square fluctuation (RMSF) analysis were performed, focusing on the enzyme’s backbone and active site. In addition, the interatomic distances between pairs of catalytic atoms were monitored throughout the simulation. Based on the RMSD of both substrate (i.e., PET dimer) and active site residues (F98-S171-M172-D217-H249), clustering analysis was performed to categorize active site conformations and thus identify the most populated clusters representing the most stable conformation of the system, using the GROMOS^28^ method in GROMACS with a cutoff of 1.9 Å and 1.2 Å, for the acylation and deacylation stages, respectively. The selected representative structures were used as a starting point for subsequent quantum mechanics/molecular mechanics molecular dynamics (QM/MM MD) simulations.

### 2.3 QM/MM MD Simulations

Molecular dynamics were performed using a hybrid QM/MM scheme as implemented in the CP2K software package.^29^ We used QUICKSTEP to calculate forces for the QM region, while FIST was used for the MM region. Density functional theory (DFT)^30^ was used for QM calculations. We used the PBE functional^31^ for the QM part with the double-ζ-valence polarization plane-wave-basis-set (DZVP-GTH-PBE) to describe valence electrons and Goedecker-Teter-Hutter (GTH) pseudopotentials for core electrons to ensure good accuracy for the generation of QM conformations under the umbrella sampling (US) protocol.^32,33^ The quality of the QM Hamiltonian in the dynamic study of enzyme-catalyzed reaction is an acknowledged pitfall of current QM/MM calculations.^34–36^ The combination of the PBE density functional with double-split valence basis sets has been widely used to study enzyme-catalyzed reactions with good accuracy.^12,34,37–40^ Other alternatives resort to the use of semiempirical QM methods for fast exploration of the conformational space, followed by free energy correction schemes at DFT level of higher levels of theory.^13,41^ Here, we have chosen to employ PBE because, although computationally expensive, it is known to systematically underestimate energy barriers by 3–5 kcal·mol^–1^, making it both consistent and predictable, and it has been shown to reproduce geometries of stationary states with good accuracy, from both benchmark studies,^37,42–45^ and free energy correction schemes.^36,41^ We set the plane-wave expansion cutoff to 300 Ry within a QM cell of dimensions 25.41 Å × 22.79 Å × 25.44 Å. For the acylation model, the QM region, which consisted of 143 atoms with a charge of −2 and singlet spin multiplicity, included parts of residues directly involved in catalysis (F98, S171, M172, D217, H249) and residues that could potentially influence it through close interactions with the substrate or catalytic residues (V218, A220, I219, V99) as well as the PET dimer substrate (Figure 4, Acylation). In the deacylation model, two water molecules were added to the QM region as they were found near S171 and H249. In addition, W170 was included as it was identified in close proximity to the substrate, increasing its potential influence on the catalysis, whereas the side chain of F98 was excluded as it was not positioned near the substrate (Figure 4, Deacylation). The QM region enclosed 133 atoms with a total charge of −1. The remaining atoms were modeled in the MM region, and MM parameters were obtained based on the previous modeling. Boundary link atoms were treated as hydrogen atoms. QM/MM long-range Coulomb interactions were included using the Gaussian expansion of the electrostatic potential (GEEP) method.^34^ Both systems were optimized with QM/MM electrostatic embedding and equilibrated with 2 ps MD in the *NVT* ensemble at 303.15 K, using the CSVR thermostat and a 1 fs integration step. Then, QM/MM steered MD simulations were performed with a harmonic potential force constant of 50 kcal·mol^–1^·Å^–2^ and a target growth of 0.0012 Å·fs^–1^, for 3 ps along the reaction coordinate for the acylation stage, and 4 ps for the deacylation stage. As reaction coordinates, we defined the following: for the acylation part (Figure 4), RC_ACYLATION_ = *d*_BREAK1_ – *d*_NUC_, where *d*_NUC_ = *d*(Oγ–C_PET_) represents the nucleophilic attack by the S171–Oγ on the carbonyl carbon of the PET dimer (C_PET_), while *d*_BREAK1_ = *d*(C_PET_-O_CPET_) refers to the cleavage of the ester bond within the PET dimer, C_PET_-O_CPET_; for the deacylation part (Figure 4), the reaction coordinate was defined as RC_DEACYLATION_ = *d*_BREAK2_ – *d*_WATER_, where *d*_BREAK2_ = *d*(Oγ–C_PET_) represents the cleavage of the Oγ–C_PET_ bond in the acylated serine, and *d*_WATER_ = *d*(O_WAT_–C_PET_) describes the nucleophilic attack by an active-site water molecule O_WAT_ on C_PET_.

**Figure 4:**
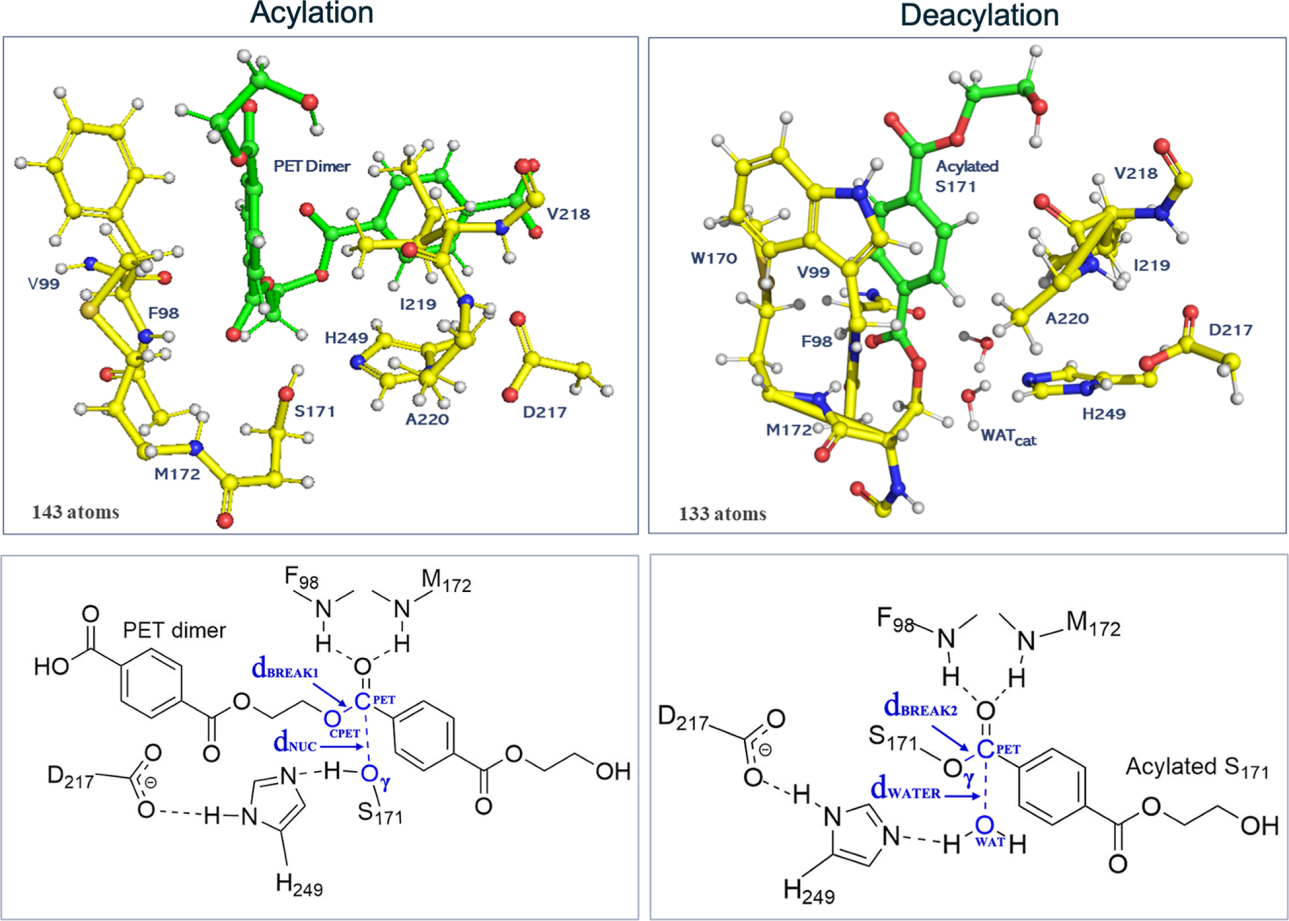
(Acylation) Top: QM layer for the acylation stage; bottom: 2D representation highlighting critical QM atoms involved in key interactions at the reactant state of the acylation stage. (Deacylation) top: QM layer for the deacylation stage; bottom: 2D representation highlighting critical QM atoms involved in key interactions at the reactant state of the deacylation stage.

### 2.4 Umbrella Sampling Simulations

Umbrella sampling simulations were run along the selected reaction coordinates to explore each mechanistic step.^46^ Based on performed steered MD simulations, a series of windows spaced at 0.1 Å intervals was generated along the defined RCs, with each window intensively sampled under an external harmonic biasing potential to ensure adequate overlap between adjacent windows (Figures S7 and S8 and Tables S3 and S4). The harmonic force constant of the biasing potential was set to 50, 100, or 200 kcal·mol^–1^·Å^–2^ in each window, according to the overlapping of the biased RC with the adjacent simulation windows (details of the bias are detailed in Table S5 of Supporting Information). Each window was simulated for 20 ps, covering 48 windows for the acylation reaction stage and 55 windows for the deacylation, resulting in a total sampling time of 2.06 ns. The unbiased free energy profile was reconstructed using the weighted histogram analysis method (WHAM).^47^ The results were presented using statistical errors estimated from 100 bootstrap data sets distributed over a bin count twice the number of windows, and a convergence tolerance of 0.0001 kcal·mol^–1^. The convergence of the free energy profiles was confirmed by ensuring adequate overlap between windows and by assessing average free energies and corresponding standard deviations across cumulative timeseries every 2 ps in forward and reverse direction, using 1 kcal·mol^–1^ as convergence threshold (block analysis every 2 ps was also conducted, Figures S9 and S10). Hence, we considered the final 16 ps per simulation for the acylation stage, and the last 12 ps for the deacylation stage (Tables S6–S9). The remaining simulation time was considered as equilibration time. To assess the nature of the maxima in each free energy profile, 2D free energy maps were determined using the WHAM-calculated Boltzmann weights for each individual conformation of the US simulations, as implemented elsewhere,^37^ and transition states were validated by ensuring that only one maximum was observed along the direction interpolated minimum free energy path (Figure S11 in Supporting Information). We present the energies in kcal·mol^–1^ with one decimal place in line with the outlined statistical criteria for convergence and given the error of the PBE/AMBER method (∼3 kcal·mol^–1^). Similarly, we present the inter- and intramolecular distances with two decimal places, which aligns with the typical accuracy of the PBE functional.

## 3 Results and Discussion

### 3.1 MD Simulations and Structural Stability

From the docking analysis, the PET dimer pose selected as optimal to assemble the PE-H/PET dimer complex was that with the lowest RMSD value relative to the reference MHET molecule and the highest docking score (Table S1 and Figure S2). Furthermore, in this solution, the distance and angle between the ester-carbon of the dimer and the hydroxyl group of catalytic S171 were 3.1 Å and 109.1°, respectively, both adequate values for a nucleophilic attack,^48^ and the oxo group of the reacting ester aligned with the backbone amine of residues F98 and M172, proposed to form a stabilizing oxyanion hole. We note that the distances between the backbone amine of F98 and M172, and the oxo of the reacting ester are 1.9 Å and 2.3 Å, supporting the viability and relevance of this docking pose (Figure S3).

The structural stability of the PE-H/PET dimer complex was also confirmed by the low and stable RMSD values observed during the 300 ns unbiased MD simulation, which fluctuate around a mean value of 1.32 ± 0.15 Å for the protein backbone and 0.71 ± 0.12 Å for the active site (Figure S4). Some variations occur during the simulation, but we can confirm that they occur outside the active site region by comparing them to the RMSD of a selection of active site residues (F98, S171, M172, D217 and H249). This is primarily attributed to the high flexibility of terminal residues as revealed by the RMSF of the backbone (Figure S5), which may be due to their interaction with the bulk water, as they are exposed to the solvent. The conservation of the catalytic geometry was assessed based on the most populated cluster (∼72% of the trajectory, determined by the RMSD of both the substrate and active site residues). The average distances between the substrate and key catalytic residues, along with their standard deviations, are reported in Table S2, demonstrating that the catalytic geometry is well preserved throughout the unbiased *NPT* simulation. Additionally, by calculating the persistence of the catalytic distances over the 300 ns MD simulation, we note that the key distances remain largely within ranges compatible with catalysis: the distance between the ester carbon of the substrate and the hydroxyl group of S171 is below 3.5 Å in 36.18% of frames; the distances between the oxo group of the reacting ester and the backbone amine of F98 and M172 are below 2.5 Å in 90.34% of frames and below 3.5 Å in 49.37% of frames, respectively. Nevertheless, when considering the full set of distances required to define productive PE-H/PET conformations, we find that only ∼12% of the simulation corresponds to productive states. The results highlight that there should be a substantial free energy penalty concerning the formation of these conformations, which will also impact on the overall free energetics of the catalysis performed by PE-H, as has been well described in recent work.^39,49,50^ Analysis of the interactions between PE-H and the PET dimer highlighted that residues F98, V99, G97, S100, W170, S171, M172, W195, D217, I219, H249, and Y250 should be involved in the binding of the dimer to the enzyme (Figure S6). These residues remain relatively stable throughout the simulation (Figure S5) and are likely involved in van der Waals forces through persistent aromatic and hydrophobic contacts. We also noted that one unit of the PET dimer exhibits a more stable interaction near the catalytic triad of PE-H, supporting findings reported by Bollinger et al.^13^ This stability might enhance the catalytic potential for hydrolysis by binding one unit in a groove adjacent to the active site like previously described molecular mechanisms.^11^

### 3.2 Reaction Mechanism—the Acylation

Using umbrella sampling simulations along the selected reaction coordinate (RC_ACYLATION_ = *d*_BREAK1_ – *d*_NUC_), we elucidated a detailed free energy profile for the acylation stage, showing two distinct transition states, the first transition state (TS1) and the second transition state (TS2), separated by a tetrahedral intermediate (TI1). The reaction occurs with free energy barriers of 7.6 and 7.5 kcal·mol^–1^ for TS1 and TS2, respectively, and a total reaction free energy of −3.7 kcal·mol^–1^. Notably, the calculated free energy barrier for the acylation stage (7.6 kcal·mol^–1^) is significantly lower than that of the overall reaction catalyzed by PETase and Cutinase (17.7 kcal·mol^–1^ and 19.0 kcal·mol^–1^, respectively),^13^ suggesting that the acylation might not be rate-limiting. Nevertheless, we also observe that formation of the reactive conformation of the reactant state is preceded by a prereactive state 0.5 kcal·mol^–1^ more stable in which the catalytic S171-hydroxyl forms an hydrogen bond with the oxo group of the PET-ester instead of the H249 (Figure S12 and Table S11). This observation suggests that formation of the reactive conformation requires a favorable preorganization of the active site and may incur a free energy cost, an hypothesis also supported by the findings of Guo et al., in which shorter distances between key catalytic residues were linked to the stabilization of transition states and could significantly lower the reaction energy barriers.^14^

In the initial reactant state R, the nucleophilic S171–Oγ is positioned within 3.06 ± 0.05 Å of the ester-carbon C_PET_. The hydrogen bonds lengths formed with F98 and M172, measuring 2.05 ± 0.23 Å and 2.89 ± 0.31 Å, contribute to hold the substrate in place for the nucleophilic attack. The acylation mechanism begins with the deprotonation of the S171-hydroxyl by the Nε atom of H249, enabling the resulting negatively charged S171–Oγ to perform a nucleophilic attack on the *sp*2-ester-carbon. At TS1, as the reaction progresses, the system undergoes significant conformational changes, with key bond distances summarized in Table S8. The S171-hydroxyl bond elongates to 1.52 ± 0.18 Å, indicating bond cleavage, while the distance from S171–Oγ to C_PET_ decreases to 1.84 ± 0.05 Å, reflecting the formation of a covalent bond between the enzyme and the substrate. In parallel, the scissile C_PET_-O_CPET_ bond elongates slightly, increasing from 1.36 ± 0.03 Å to 1.42 ± 0.04 Å, and H249 becomes protonated (Nε–H distance shortens to 1.13 ± 0.10 Å, confirming the proton transfer). At TI1, the proton transfer to H249 is concluded (1.06 ± 0.04 Å) and the tetrahedral intermediate is formed (S171Oγ–C_PET_ shortens to 1.51 ± 0.03 Å, Figure 5c). The oxyanion hole, formed by F98 and M172, played a crucial role in stabilizing the TI1 by maintaining hydrogen bonds with the evolving intermediate (Figure 5c). This stabilization is particularly evident as the reaction progresses from TS1 to TI1 and onward to TS2 (Figure 6), as the free energy required to weaken these hydrogen bonds becomes higher at this point (>5 kcal·mol^–1^, as opposed to the <1 kcal·mol^–1^ required at the reactant, Figure S14 in Supporting Information). Proton transfer events between H249 and D217 also become barrierless as TI1 is formed (Figure S13 in Supporting Information), in close agreement with literature on the contribution of low barrier hydrogen bonds to the catalysis by serine esterases and proteases exhibiting the S–H-D triad.^51^

**Figure 5:**
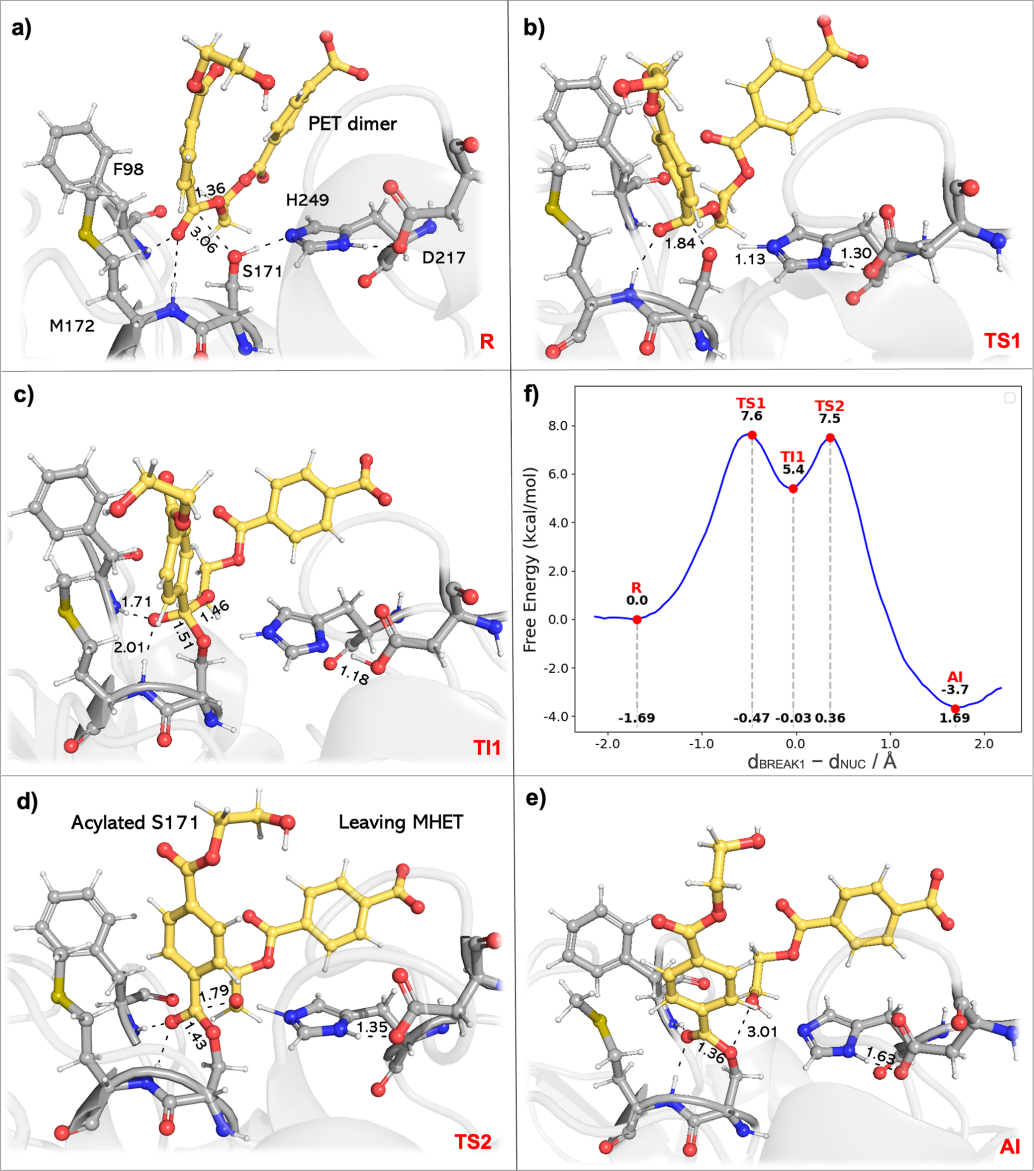
Free energy profile and representative structures along the acylation reaction pathway of PE-H. The reaction pathway proceeds from (a) the reactant state (R) through (b) the first transition state (TS1), characterized by the nucleophilic attack of the S171-hydroxyl on the ester-carbon C_PET_, concomitant with the deprotonation S171-hydroxyl by the Nε of H249, followed by (c) the tetrahedral intermediate (TI) exhibiting an oxyanion hole formed between the backbone amine of F98 and M172 and the formed C_PET_–alkoxide (O_CPET_). Subsequent progression involves (d) the second transition state (TS2), in which there is C_PET_-O_CPET_ bond cleavage and MHET release, culminating in (e) the acyl-enzyme intermediate (AI). (f) The corresponding free energy profile for this acylation stage displays free energy barriers and the reaction free energy (the reactant state set to 0.0 as the reference) with corresponding reaction coordinates (gray dotted line). Relevant distances are given in Å.

**Figure 6:**
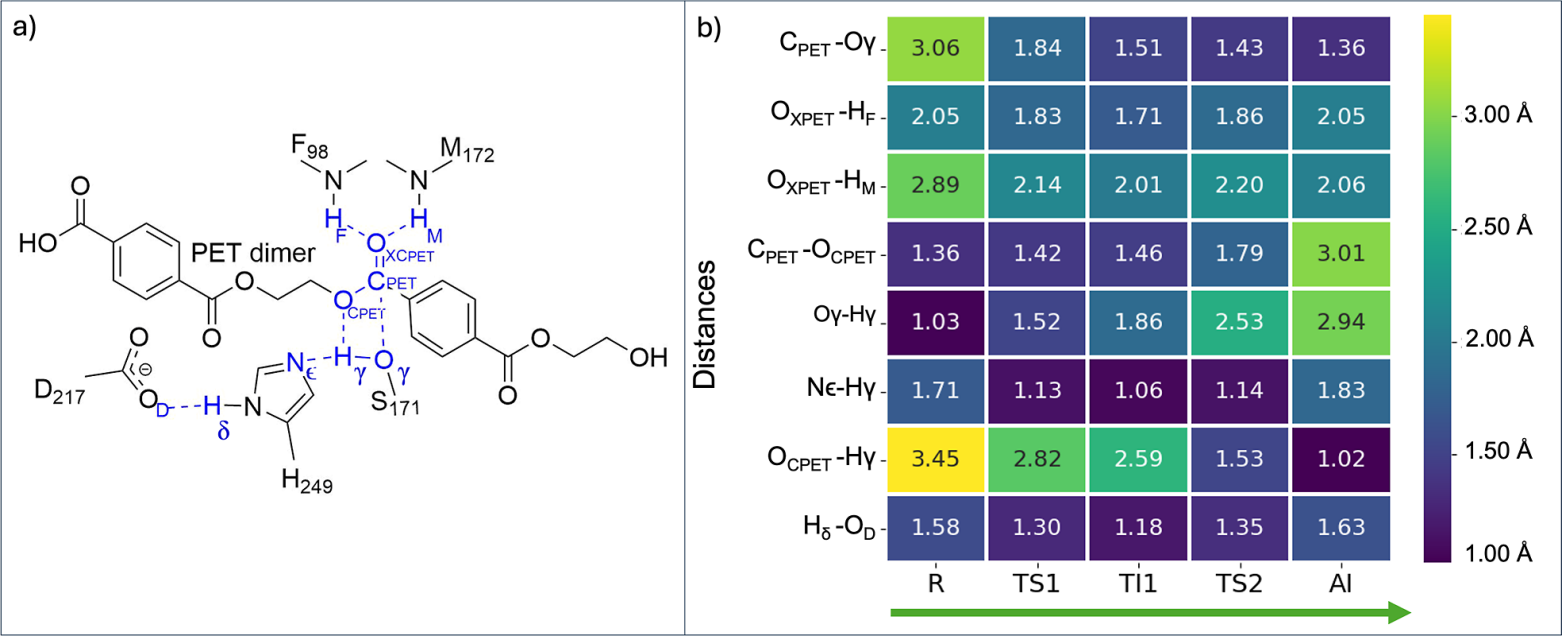
a) 2D scheme highlighting critical atoms involved in key interactions. (b) Comparative heatmap of key interatomic average distances (given in Å) across reaction states (R → TS1 → TI1 → TS2 → AI). Standard deviations are provided in the Supporting Information (Table S10).

Following the formation of TI1, the reaction proceeds to TS2, where the C_PET_-O_CPET_ scissile bond breaks completely. During this step, the tetrahedral intermediate is resolved as the C_PET_-O_CPET_ bond elongates to 1.79 ± 0.05 Å, leading to the release of MHET and the formation of the acyl-enzyme complex with S171 (Figure 5d). Following this cleavage, the positively charged H249 is deprotonated by the leaving group (MHET), with the distance between the S171–Hγ and O_CPET_ decreasing to 1.02 ± 0.04 Å. The free energy barrier (7.6 kcal·mol^–1^) for TS1 is higher than that of TS2, yielding TS1 the rate-limiting step for the acylation. The oxyanion hole continues to play a critical role in stabilizing TS2 as the reaction proceeds toward the acylation intermediate (AI), where MHET is released, as confirmed by the significant increase of the C_PET_-O_CPET_ distance to 3.01 ± 0.02 Å. Concurrently, the bond between the S171–Oγ and C_PET_ shortens to 1.36 ± 0.03, evidencing the formation of a C–O single bond.

While experimental data for PE-H are limited, the observed stabilization by the oxyanion hole (F98/M172 NH groups) aligns with mechanisms reported for PETase and Cutinase.^13^ The heatmap (Figure 6b) reveals progressive decrease of oxyanion hole distances, suggesting a strengthening of these interactions throughout the reaction. During TS1 formation the distances between F98/M172 NH groups and the ester-oxo group decrease from 2.05 to 1.83 Å and 2.89 to 2.14 Å, respectively. This stabilization is maintained throughout TI1 and TS2, and a final relaxation occurs in the AI state (distances increase to 2.05 and 2.06 Å).

This pattern confirms the oxyanion hole’s critical role in stabilizing the tetrahedral transition state and subsequent intermediates. Consistent with these observations, the heatmap highlights anticorrelated distance changes for nucleophilic bond formation (S171Oγ–C_PET_ decrease: 3.06 Å–1.36 Å) and substrate cleavage (C_PET_–O_CPET_ elongation: 1.36 Å–3.01 Å), revealing reciprocal distance changes and demonstrating the concerted attack-and-cleavage mechanism, a pattern that correlates with PETase’s and Cutinase’s catalytic strategies.^12,13^ The results on the stabilization by the oxyanion hole are also in agreement with other works where the stabilizing effect of the oxyanion hole was tested and extensively discussed, namely by performing substitution of the hydrogen bond donor amide groups by a methylene group.^52–54^

Subsequent conventional MD simulations of 100 ns showed MHET rapidly dissociating (<0.4 ns) and being replaced by bulk water. This indicates that MHET diffusion to the bulk is likely a spontaneous, entropy-driven process. Comparative analysis of the acylation free energy profiles of PE-H with PETase and Cutinase,^12,13^ reveals that PE-H exhibits significantly lower free energy barriers, which may imply a more efficient reaction pathway. In particular, Jerves et al.^12^ identified a single, concerted transition state (TS1, 20.0 kcal·mol^–1^, calculated at the PBE/MM level at 300 K) for PETase acylation, whereas PE-H exhibits a stepwise mechanism with two distinct transition states, the first one being rate-limiting (TS1, 7.6 kcal·mol^–1^, calculated at the PBE/MM level at 303.15 K), suggesting a critical evolutionary trade-off: whereas PETase prioritizes catalytic rate through a preorganized, concerted mechanism, PE-H appears to sacrifice barrier height for precise intermediate stabilization via its stepwise pathway.^12^ This difference could be structurally rooted in the shorter PE-H Ser-Oγ–C_PET_ distance (3.06 ± 0.05 Å) compared to PETase (3.30 ± 0.14 Å) and the optimized hydrogen bond distances of the oxyanion hole (2.05 ± 0.23 Å and 2.89 ± 0.31 Å for PE-H, and 2.68 ± 0.57 Å and 3.07 ± 0.44 Å for PETase).

### 3.3 Reaction Mechanism—the Deacylation

During the deacylation stage, a water molecule from the active site (WAT_cat_) diffused from the bulk to occupy the space previously occupied by MHET, readying the acyl-enzyme intermediate complex for the subsequent deacylation stage. The outcome of such catalytic step led to product formation and regenerated the enzyme to its resting state. The starting configuration of the deacylation stage (AI) does not change significantly despite MHET exiting the active site after ∼0.4 ns. Molecular dynamics simulations revealed a high presence of water molecules in the region, with water molecules present in the proximity of the catalytic atoms in around 46% of the 100 ns simulations (based on selecting frames where water was within 3 Å of the H249’s Nε and 5 Å of the C_PET_), supporting the occupation of the active site region by water.

The free energy profile for the deacylation process (Figure 7f) revealed three key intermediates: the acylation intermediate state (AI), the tetrahedral intermediate (TI2), and the final product (PC), bridged by two transition states (TS3 and TS4). This stage had a maximum activation barrier of 10.6 kcal·mol^–1^.

**Figure 7:**
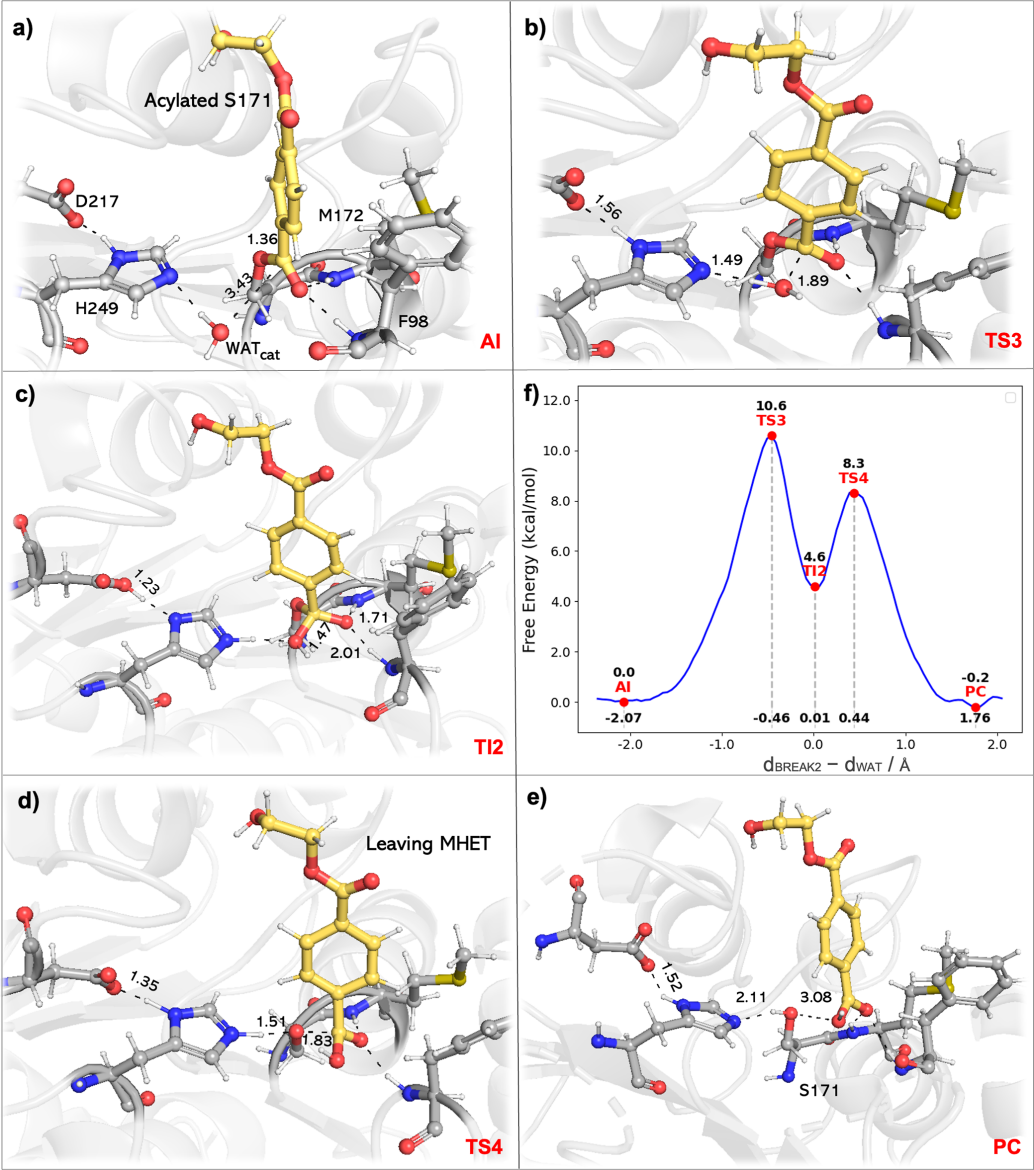
Free energy profile and representative structures along the deacylation reaction pathway of PE-H. The reaction proceeds from (a) the intermediate state (AI) through (b) the first transition state (TS3), characterized by the nucleophilic attack by the catalytic water (WAT_cat_) on the acylated S171, concomitant with the deprotonation of WAT_cat_ by the Nε of H249. (c) A tetrahedral intermediate (TI2) is subsequently formed, stabilized within the oxyanion hole formed between the backbone amine of F98 and M172, and featuring a C_PET_–alkoxide (O_XPET_) center. Subsequent progression involves (d) the second transition state (TS4), which is associated with C_PET_–Oγ bond cleavage and MHET release, culminating in (e) the product state (PC). (f) The corresponding free energy profile for this deacylation stage displays the free energy barriers and the reaction free energy (the reactant state set to 0.0 as the reference) with corresponding reaction coordinates (gray dotted line). Relevant distances are given in Å.

In the initial AI state, the close proximity between H249’s Nε and the WAT_cat_ (1.86 ± 0.32 Å) facilitates water activation via deprotonation. Concurrently, the distance between the WAT_cat_ oxygen and the C_PET_ of S171 (3.43 ± 0.05 Å) is well-suited to enable nucleophilic attack (Figure 7a). As the reaction progresses toward the first transition state (TS3), key geometric rearrangements occur (Table S12). Notably, the distance between the O_wat_ and the carbonyl carbon of the acylated S171 decreases sharply to 1.89 ± 0.05 Å, facilitating the nucleophilic attack by the O_wat_ (Figure 7b). Simultaneously, the deprotonation of WAT_cat_ by the Nε of H249 is initiated (Nε-H_wat_ distance decreases from 1.86 ± 0.32 Å to 1.49 ± 0.24 Å), indicating simultaneous nucleophilic attack and deprotonation reactions during the TS3 formation.

Following TS3, the reaction proceeds to the formation of the tetrahedral intermediate (TI2), characterized by a fully formed covalent bond between the O_wat_ and the acyl carbon C_PET_ (1.47 ± 0.04 Å). This configuration adopts a tetrahedral geometry analogous to that observed in the TI1 of the acylation stage. At this stage, the proton is effectively transferred from WAT_cat_ to H249 (1.06 ± 0.04 Å). Simultaneously, the resulting oxyanion intermediate is stabilized through hydrogen bonds established with the oxyanion hole-forming residues F98 and M172 (1.64 ± 0.14 and 1.85 ± 0.18 Å, respectively). The stabilization by the oxyanion hole is, nevertheless, less pronounced than for the acylation step (Figure S14 in Supporting Information), which is consistent with reports in the literature.^12^ As for the acylation step, proton transfer events between H249 and D217 are again observed, although the acid form of D217 becomes slightly more prevalent once the TI2 is formed (Figure S13 in Supporting Information).

The reaction then advances toward TS4. Here, the collapse of the tetrahedral intermediate is marked by the elongation of the bond between the acyl carbon C_PET_ and the oxygen Oγ, which increases from 1.49 ± 0.04 Å to roughly 1.83 ± 0.05 Å, indicating bond cleavage (Figures 7d and 8). Following this cleavage and the release of the second MHET unit, deprotonation of H249 by the negatively charged S171 rapidly follows, leading to the product state (PC), which confirms the concerted asynchronous nature of the reaction (Figure 7d,e). Following deacylation, the product is no longer covalently bound, and the active site becomes more solvent-exposed. The oxyanion hole stabilizing interactions are weakened (1.91 ± 0.18 and 2.39 ± 0.30 Å) at this stage, consistent with the structural features associated with the departure of MHET after the acylation step. The product is therefore expected to dissociate readily.

**Figure 8:**
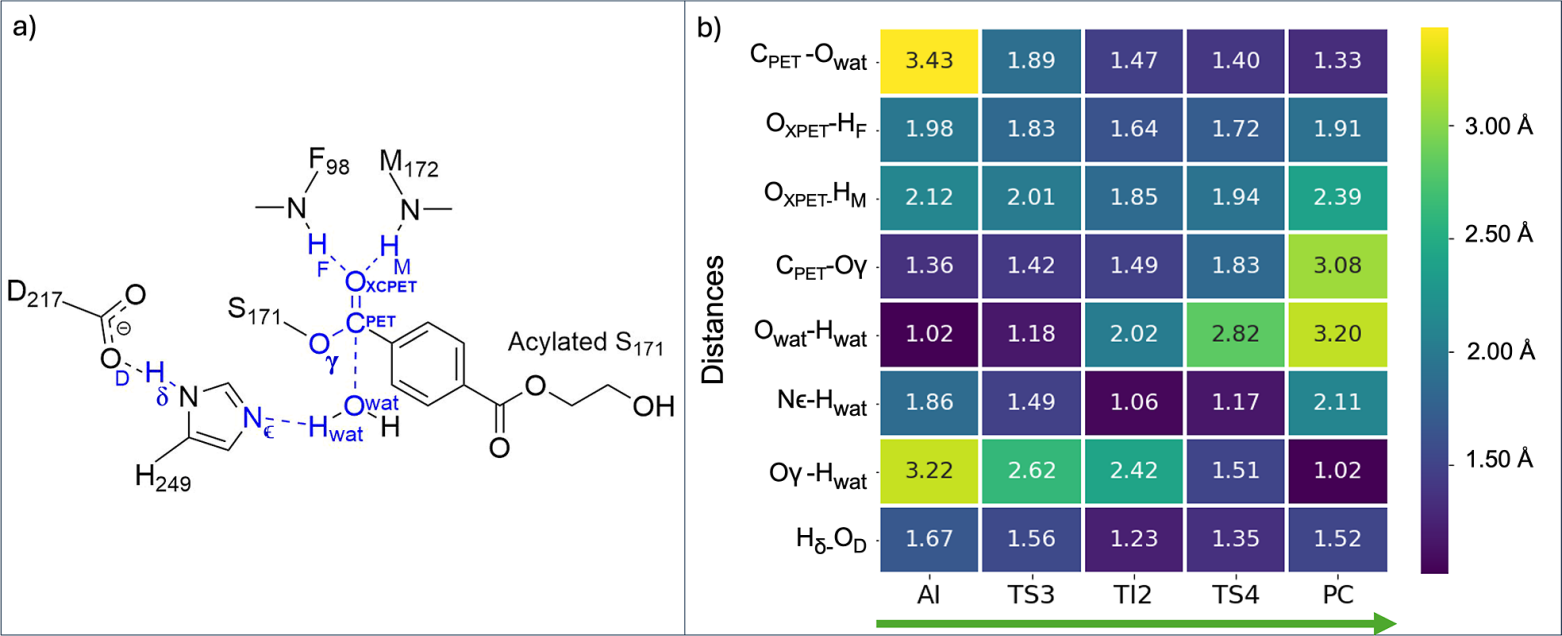
(a) 2D scheme highlighting critical atoms involved in key interactions. (b) Comparative heatmap of key interatomic distances across reaction states (R → TS3 → TI2 → TS4 → PC) of key interatomic distances (Å). Standard deviations are provided in the Supporting Information (Table S12).

This mechanism aligns with the acylation stage mechanism, where the proton transfer (*i.e.*, activation of the nucleophile) and nucleophilic attack precede bond cleavage. Notably, while both acylation and deacylation stages involve the formation of a tetrahedral intermediate, the nucleophile differs: S171 in acylation vs the WAT_cat_ in deacylation. Both stages are defined by two transition states, underscoring their stepwise nature. Our calculations indicate that TS3 in the deacylation stage may be the rate-limiting step of the overall reaction. Nevertheless, since the highest activation free energies for both stages (TS1 and TS3) only differ in about 3 kcal·mol^–1^, we emphasize that the free energetics for the formation of reactive conformations might also impact whether the acylation or deacylation are rate-limiting. These transition states, stabilized by the oxyanion hole, also reflect the conserved nature of the catalytic mechanism while highlighting the differences in energetic profiles that influence enzyme efficiency.

Our findings follow several studies supporting that deacylation can be the rate-limiting step in PET hydrolysis.^14,55,56^ The study by Guo et al. or the one by Magalhães et al. demonstrates a stepwise mechanism with deacylation as the rate-limiting step.^14,57^ However, while employing the same PBE/MM MD approach, Jerves *et al*. proposed a concerted mechanism with acylation as the rate-limiting step.^12^ The difference in results may come from PE-H’s shorter catalytic distances when compared to those observed for PETase, which could enable more precise stabilization of the tetrahedral intermediate and facilitate the observed stepwise process. This reveals that the PE-H catalytic reaction may be expected to proceed more effectively as a result of favorable interactions and an optimal active site geometry, ultimately lowering the overall energy barriers necessary for the reaction to take place. The observed differences in transition-state processes suggest that distinct active-site configurations may correlate with alternative rate-limiting steps in PET-hydrolyzing enzymes, although further studies are needed to establish causative relationships. PE-H also exhibits a lower pI than PETase (6.54 vs 9.28) and different composition in charged residues (24 negatively charged and 22 positively charged residues, as opposed to 13 and 19 for PETase), although the local electrostatic environment of both enzymes is similar, and thus we cannot exclude that distal electrostatic effects might also contribute for its efficiency.

## 4 Conclusions

Polyester hydrolase (PE-H) from the marine bacterium *P. aestusnigri* has emerged as a promising biocatalyst for the enzymatic depolymerization of polyethylene terephthalate (PET). Herein, we present a detailed atomistic characterization of the PE-H catalytic cycle, elucidated via a combination of advanced quantum mechanics/molecular mechanics modeling (PBE/AMBER) and umbrella sampling simulations, employing a PET dimer model substrate. Our study reveals a stepwise process where the acylation stage is characterized by an initial transition state (TS1) at a free energy barrier of 7.6 kcal·mol^–1^, followed by a subsequent transition state (TS2) at 7.5 kcal·mol^–1^, corresponding to the conversion of the tetrahedral intermediate to the acyl-enzyme intermediate complex. Consistently, deacylation, emerging as the rate-limiting step, presents a higher initial barrier, with TS3 occurring at 10.6 kcal·mol^–1^, followed by a second transition state (TS4) at 8.3 kcal·mol^–1^, associated with hydrolysis of the acyl-enzyme intermediate and product release, respectively. Although this model differs from Jerves *et al*.’s concerted process and higher barrier energy under identical PBE level, the observed variation in outcome may be attributed to PE-H’s shorter catalytic distances and oxyanion hole, which could potentially enable more precise stabilization of the tetrahedral intermediate and facilitate the observed stepwise process. This is consistent with the two-step process of Guo *et al.*, which showed a lower barrier and suggested that shorter distances between key catalytic residues are associated with the stabilization of transition states and could significantly lower reaction energy barriers.

Following analogous pathways to classic serine hydrolases, the catalytic triad functions through a sophisticated mechanism: S171 acts as nucleophile, H249 serves as general base for proton transfer, while D217 synergistically supports proton transfer and stabilization. Both transition states receive substantial stabilization from the oxyanion hole formed by F98 and M172 backbone amines. These critical hydrogen bonds remain intact throughout the reaction cycle and reach optimal geometry in all transition states, playing a pivotal catalytic role.

Our results support a general proposal in which the PE-H catalytic reaction may be expected to proceed more effectively due to favorable interactions and optimal active site geometry, reducing the energy barriers for the reaction. These findings provide both fundamental mechanistic insights into PE-H catalysis and practical guidelines that can likely be extended to other enzymes in the PET-degrading family, bringing us closer to practical large-scale PET waste bioremediation solutions.

## Supplementary Material







## Data Availability

The Supporting Information includes topology and coordinate files for the solvated PEH:PET complex, provided in machine-readable formats for both GROMACS 2021.3 (Gmx-md_inputs.zip). Molecular docking was carried out using GOLD Suite 5.2.2 with the ChemPLP scoring function, available under the CCDC License Agreement for Paid Products (https://www.ccdc.cam.ac.uk/licence-agreement/). Molecular mechanics MD simulations were conducted using GROMACS 2021.3, which is freely accessible at https://manual.gromacs.org/documentation/2021.3. Data collection and analysis were performed using the trjconv, rms, rmsf, distance, and cluster modules of GROMACS 2021.3. QM/MM simulations were carried out using CP2K 8.2, open and freely available for everybody under the GPL license at https://manual.cp2k.org, and the sander module of the AMBER 18 package, also available free of charge in the AmberTools 18 suite upon registration at https://ambermd.org/GetAmber.php#ambertools. The AMBER package is licensed software that can be purchased for both academic and industrial applications (https://ambermd.org). Input files required to reproduce the QM/MM simulations are provided in the Supporting Information (Cp2k-amber-qmmm-md_inputs.zip). Data collection and analysis of the QM/MM simulations were performed with the CPPTRAJ module included in the AMBER 18 and AmberTools 18 packages. Free energy profiles from umbrella sampling simulations were obtained using WHAM 2.0.11 (http://membrane.urmc.rochester.edu). Analysis workflows were automated using internally developed Python 3.8.10 (https://www.python.org/downloads/release/python-3810) and Bash (.sh) scripts, employing the modules pandas 2.0.3 (https://pypi.org/project/pandas/2.0.3), numpy 1.24.4 (https://pypi.org/project/numpy/1.24.4), and matplotlib 3.7.5 (https://matplotlib.org/3.7.5/contents.html). Structural preparation and inspection of docking poses and MD/QM/MM trajectories were carried out with VMD 1.9.4a55, freely available for academic use at https://www.ks.uiuc.edu/Research/vmd/, and with PyMOL 2.3.0, also available at https://pymol.org. Visualization and figure preparation were also performed with PyMOL 2.3.0.

## Notes

Description of the energy minimization protocol for geometric optimization of the PE-H:PET complex. Optimal docking solutions for the PE-H:PET complex. PET dimer binding to the active site of PE-H. RMSD of the protein and active site during the 300 ns MD simulation. RMSF variation throughout the 300 ns MD simulation. The representative structure of PET dimer binding site highlighting key interacting residues. Average free energies by cumulative forward and reverse timeseries obtained across reaction states for the acylation and deacylation stages. Average distances across reaction states for acylation and deacylation stages. The corresponding free energy profile for acylation stage displaying a prereactive conformation at the beginning of the reaction. Average distances obtained for the prereactive stage for the acylation. Average distances obtained for the prereactive stage for the acylation (PDF)
GROMACS input files for energy minimization and MD simulations (ZIP)
CP2K input files for QM/MM energy minimization, equilibration, steered and umbrella sampling MD simulations (ZIP)
