# Supplementary material for: Mechanism of Polyester Hydrolysis by Marine Bacterium PE‑H Enzyme: an Atomistic and Thermodynamic Characterization
Source: J Chem Inf Model. 2026 Feb 12;66(4):2284–98. doi: 10.1021/acs.jcim.5c02314 (PMC12933714; doi:10.1021/acs.jcim.5c02314)
Supplement: Supplementary file 1 [file ci5c02314_si_001.pdf]

Supporting Information

On the mechanism of polyester hydrolysis by marine bacterium PE-H enzyme: an atomistic and thermodynamic characterization

Samah Nassir[a,b], Pedro Paiva[a], Rui P. P. Neves[a], Pedro A. Fernandes[a], Achraf El Allali[b], Maria J. Ramos[a]**

[a] LAQV/REQUIMTE, Departamento de Química e Bioquímica, Faculdade de Ciências Universidade do Porto, Rua do Campo Alegre, s/n, 4169-007 Porto, Portugal

[b] Bioinformatics Laboratory, College of Computing, University Mohammed VI Polytechnic, Lot 660, Hay Moulay Rachid, Ben Guerir 43150, Morocco

*corresponding authors: Achraf.ELALLALI@um6p.ma and mjramos@fc.up.pt

Energy minimization protocol for geometric optimization of the PE-H:PET complex

For the assembled molecular system, a five-step minimization protocol was performed to allow the system to adapt to the modeling procedure using an energy step size of 0.01 kJ/mol and a convergence criterion for the maximum force of 1000 kJ/mol.

1) Minimization of water molecules and counterions while restraining all the rest of the system.
2) Minimization of all hydrogen atoms, keeping the rest of the system constrained.
3) Optimization of the side chains of the protein, with positional restraints applied to the remaining system.
4) Relaxation of the system while maintaining restraints on the backbone amide residues that form the oxyanion hole, as well as the carbonyl group of the substrate.
5) Minimization of the whole system with no applied constraints.

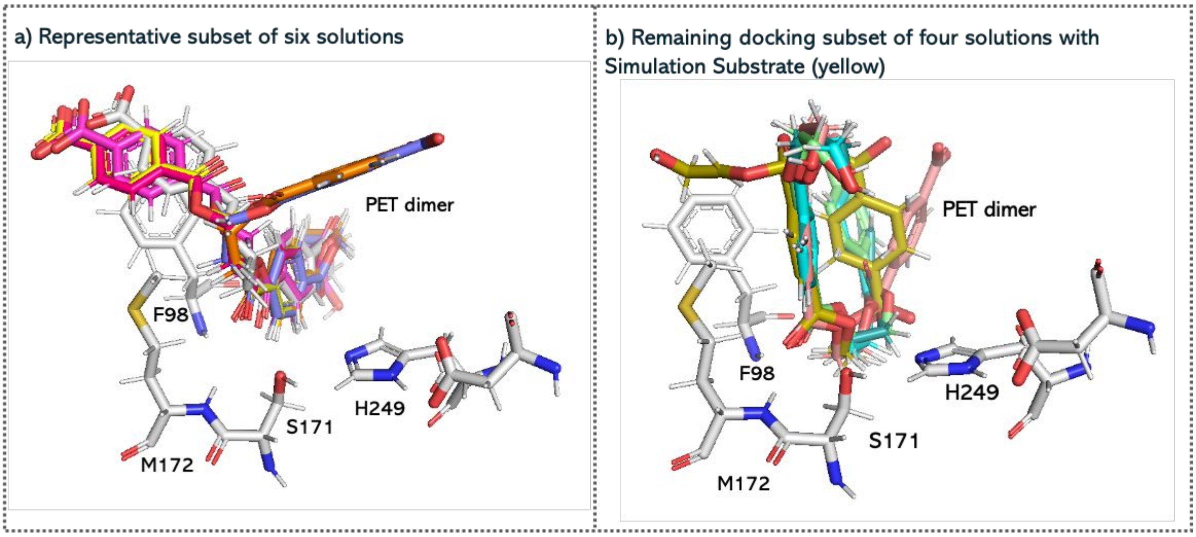

Figure S1: Docking results obtained from the unconstrained docking run for the PET dimer relative to the catalytic S171, H249, F98 and M172 residues. All ten generated solutions are included: a) A subset of the first six poses with similar binding positions and interactions. b) The remaining four poses together with the substrate used for subsequent simulations, highlighting their relative alignment and spatial distribution.

Table S1: Docking solutions sorted by Fitness with RMSD values given in Å. Grey shading indicates the selected solution.

Index	PLP.Fitness	PLP.REFERENCE. RMSD
**5**	59.8764	3.0767
**1**	58.3983	2.7643
**2**	57.3720	2.6587
**4**	57.2428	3.1079
**3**	56.4255	4.5847

**9**	56.0505	2.1976
**10**	54.7620	2.0359
**7**	54.3471	4.7318
**8**	53.9724	4.7476
**6**	53.2879	4.6063

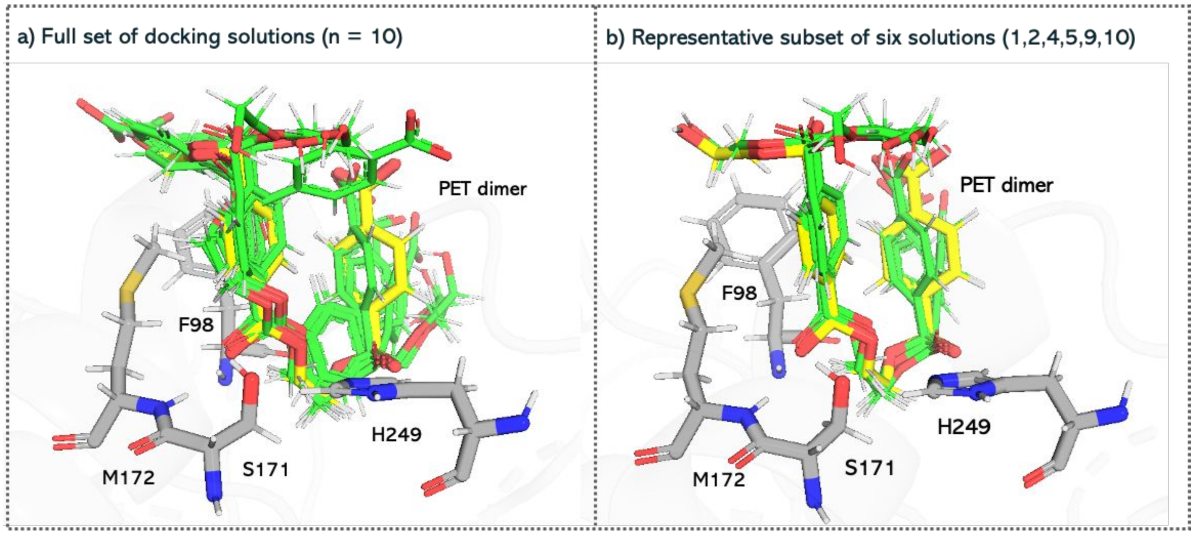

Figure S2: Superposition of docking solutions for the PET dimer relative to the catalytic S171, H249, F98 and M172 residues. The solution used for the subsequent simulations is highlighted in yellow, and the remaining solutions are shown in green. a) All ten poses generated by docking show a highly conserved orientation of the PET dimer with respect to the catalytic residues. b) A subset of six solutions illustrates the minimal structural variation among poses. In all solutions, the key distances between the substrate and the catalytic residues fall within a narrow range, indicating strong convergence of the docking procedure and supporting the reliability of the selected starting configuration for the MD simulations.

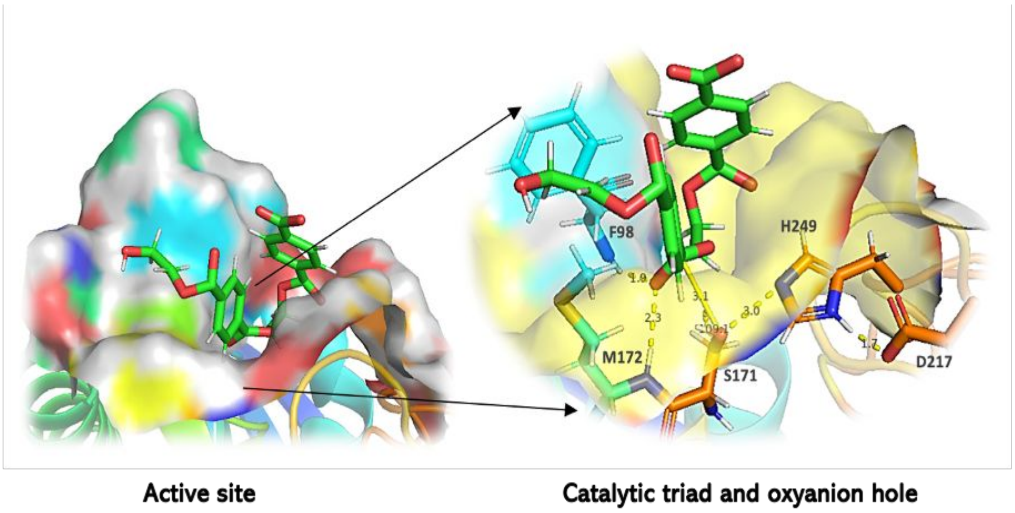

Figure S1: PET dimer binding to the active site stabilized by the oxyanion hole (backbone amides of residues F98 and M172). The catalytic S171 adopts an adequate position to perform an attack on the substrate's (green sticks) carbonyl carbon for ester hydrolysis. The surface cleft is lined by F98, S100, W170, M172, W195, I219, H249, and Y250. We noted the same residues aligned to the surface cleft of

the experimental substrate from the ICCG variant of the leaf branch compost Cutinase structure (PDB ID: 7vve).[1]

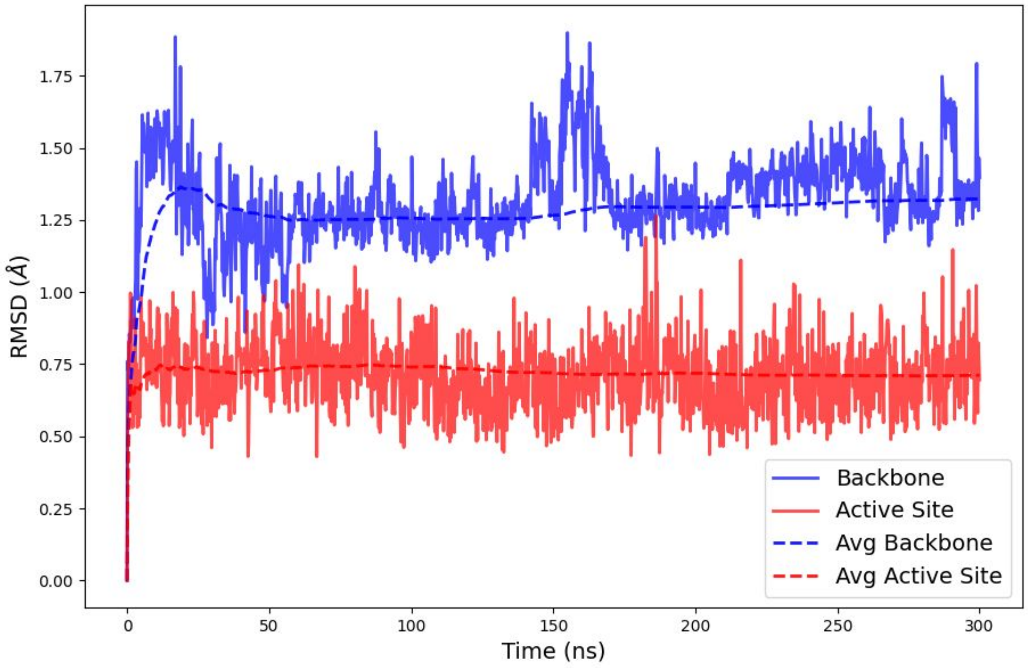

Figure S2: Root mean square deviation (RMSD) during the 300 ns MD simulation, using the first structure as a reference. The blue line corresponds to the protein backbone, and the blue line corresponds to the active site. The accumulated average is shown as blue and red lines. The accumulated averages show that the system is converged.

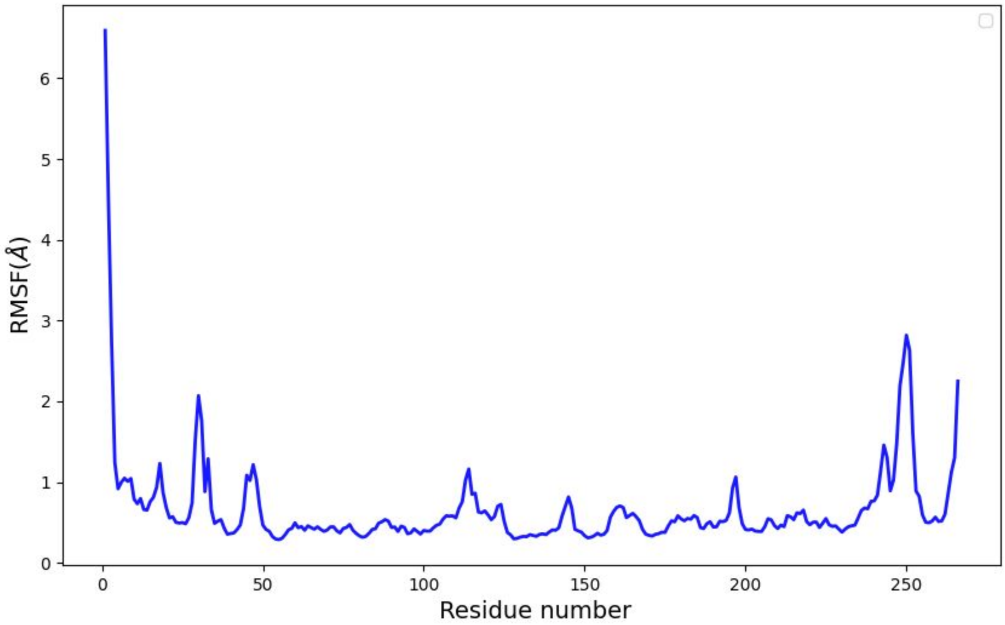

Figure S3: Protein residues root-mean-square fluctuation (RMSF) variation throughout the 300 ns MD simulation. The terminal residues are more flexible compared to the rest of the residues due to their interaction with the bulk water, as they are abundantly exposed to the solvent.

Table S2: Average distances between the substrate and key catalytic residues, along with their standard deviations (given in Å) from the representative cluster.

Distance	Average	Std
**S171Oγ-CPET**	3.61	0.22
**OXPET-F98NH**	2.13	0.28
**OXPET-M172NH**	3.54	0.45
**CPET-OCPET**	1.36	0.00
**S171Oγ-Hγ**	0.96	0.00
**H249Nϵ-S171Hγ**	2.53	0.57
**OCPET-S171Hγ**	2.83	0.14

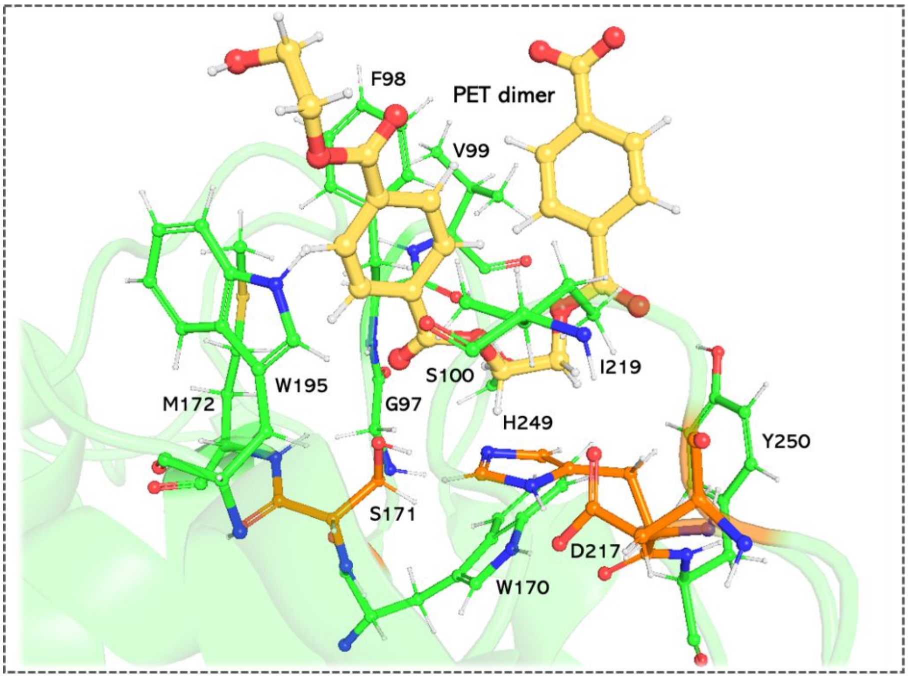

Figure S6: The representative structure of PET dimer binding site highlighting key interacting residues. Contacting residues (identified using a 5 Å cutoff between any protein atoms and substrate atom) are shown in green sticks, while catalytic residues are shown in orange.

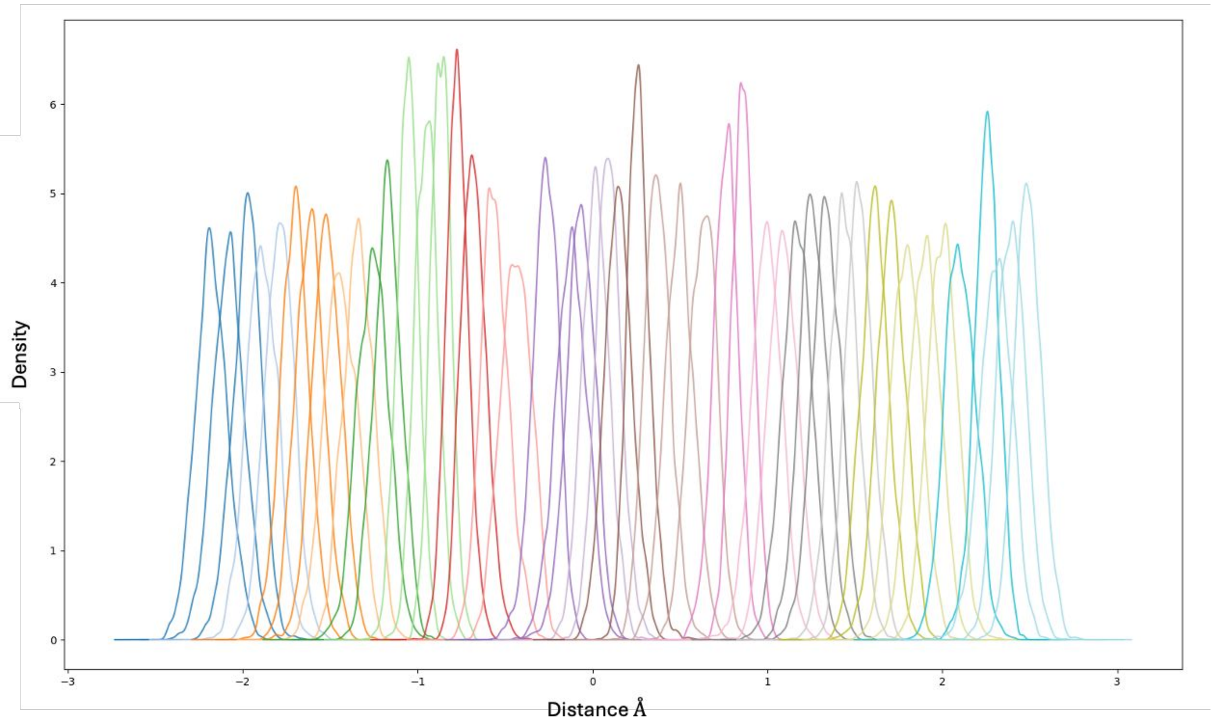

Figure S7: Density vs. umbrella sampling window for the reaction coordinate $d_{break1} - d_{nuc}$.

Table S3: The histogram distribution for the umbrella sampling windows of the acylation stage.

RC_i / Å	min	Q1	median	Q3	max	mean	stdev	mean-ref	Q1-to-mean	Q3-to-mean
**-2.20**	-2.45	-2.24	-2.18	-2.12	-1.88	-2.18	0.09	0.02	0.06	0.06
**-2.10**	-2.42	-2.14	-2.08	-2.02	-1.79	-2.08	0.09	0.02	0.06	0.06
**-2.00**	-2.27	-2.03	-1.97	-1.92	-1.65	-1.98	0.08	0.02	0.05	0.06
**-1.90**	-2.22	-1.95	-1.89	-1.83	-1.58	-1.89	0.09	0.01	0.06	0.06
**-1.80**	-2.06	-1.85	-1.79	-1.73	-1.51	-1.79	0.08	-0.01	0.06	0.06
**-1.70**	-2.01	-1.76	-1.70	-1.65	-1.44	-1.70	0.08	-0.00	0.05	0.05
**-1.60**	-1.88	-1.67	-1.62	-1.56	-1.36	-1.62	0.08	-0.02	0.06	0.06
**-1.50**	-1.85	-1.58	-1.52	-1.46	-1.24	-1.52	0.08	-0.02	0.06	0.06
**-1.40**	-1.75	-1.51	-1.44	-1.38	-1.18	-1.44	0.09	-0.04	0.06	0.07
**-1.30**	-1.59	-1.40	-1.34	-1.28	-1.02	-1.34	0.09	-0.04	0.06	0.06
**-1.20**	-1.57	-1.32	-1.26	-1.20	-0.95	-1.26	0.09	-0.06	0.06	0.06

**-1.10**	-1.45	-1.23	-1.17	-1.12	-0.91	-1.17	0.08	-0.07	0.05	0.05
**-1.00**	-1.30	-1.10	-1.05	-1.01	-0.85	-1.06	0.06	-0.06	0.04	0.04
**-0.90**	-1.18	-1.00	-0.95	-0.91	-0.74	-0.95	0.07	-0.05	0.05	0.04
**-0.80**	-1.04	-0.91	-0.87	-0.83	-0.68	-0.87	0.06	-0.07	0.04	0.04
**-0.70**	-1.03	-0.82	-0.77	-0.73	-0.56	-0.77	0.06	-0.07	0.04	0.04
**-0.60**	-0.89	-0.73	-0.68	-0.63	-0.36	-0.68	0.07	-0.08	0.05	0.05
**-0.50**	-0.80	-0.61	-0.56	-0.50	-0.28	-0.56	0.08	-0.06	0.05	0.05
**-0.40**	-0.71	-0.50	-0.44	-0.38	-0.18	-0.44	0.09	-0.04	0.06	0.06
**-0.30**	-0.58	-0.32	-0.27	-0.22	-0.05	-0.27	0.08	0.03	0.05	0.05
**-0.20**	-0.44	-0.18	-0.12	-0.06	0.19	-0.12	0.09	0.08	0.06	0.06
**-0.10**	-0.41	-0.12	-0.07	-0.01	0.19	-0.07	0.08	0.03	0.05	0.06
**0.00**	-0.27	-0.04	0.02	0.07	0.32	0.02	0.08	0.02	0.05	0.05
**0.10**	-0.16	0.04	0.09	0.14	0.36	0.09	0.07	-0.01	0.05	0.05
**0.20**	-0.09	0.09	0.15	0.20	0.40	0.15	0.08	-0.05	0.05	0.05
**0.30**	0.03	0.22	0.26	0.30	0.59	0.26	0.07	-0.04	0.05	0.04
**0.40**	0.12	0.32	0.37	0.42	0.66	0.37	0.08	-0.03	0.05	0.05
**0.50**	0.25	0.44	0.50	0.55	0.79	0.50	0.08	-0.00	0.06	0.06
**0.60**	0.34	0.57	0.63	0.69	0.90	0.63	0.08	0.03	0.06	0.06
**0.70**	0.53	0.71	0.76	0.81	1.01	0.76	0.07	0.06	0.05	0.05
**0.80**	0.64	0.82	0.86	0.90	1.07	0.86	0.06	0.06	0.04	0.04
**0.90**	0.61	0.94	0.99	1.05	1.28	0.99	0.09	0.09	0.06	0.06
**1.00**	0.75	1.03	1.09	1.15	1.37	1.09	0.09	0.09	0.06	0.06
**1.10**	0.86	1.11	1.17	1.23	1.44	1.17	0.09	0.07	0.06	0.06
**1.20**	1.00	1.21	1.26	1.31	1.52	1.26	0.08	0.06	0.05	0.06
**1.30**	1.04	1.28	1.33	1.39	1.60	1.33	0.08	0.03	0.05	0.06
**1.40**	1.13	1.38	1.44	1.49	1.74	1.44	0.08	0.04	0.06	0.05
**1.50**	1.25	1.47	1.52	1.58	1.77	1.52	0.08	0.02	0.05	0.05
**1.60**	1.35	1.56	1.61	1.67	1.93	1.61	0.08	0.01	0.05	0.05
**1.70**	1.37	1.65	1.71	1.76	1.97	1.71	0.08	0.01	0.06	0.06
**1.80**	1.49	1.73	1.79	1.85	2.07	1.79	0.09	-0.01	0.06	0.06
**1.90**	1.58	1.84	1.90	1.96	2.18	1.90	0.09	-0.00	0.06	0.06
**2.00**	1.64	1.94	2.00	2.06	2.34	2.00	0.09	0.00	0.06	0.06
**2.10**	1.81	2.04	2.10	2.16	2.42	2.10	0.09	0.00	0.06	0.06
**2.20**	2.00	2.21	2.25	2.30	2.47	2.25	0.07	0.05	0.04	0.05
**2.30**	1.96	2.24	2.31	2.37	2.61	2.31	0.09	0.01	0.06	0.06
**2.40**	2.08	2.34	2.39	2.45	2.72	2.39	0.08	-0.01	0.06	0.06
**2.50**	2.22	2.44	2.49	2.54	2.79	2.49	0.08	-0.01	0.05	0.05

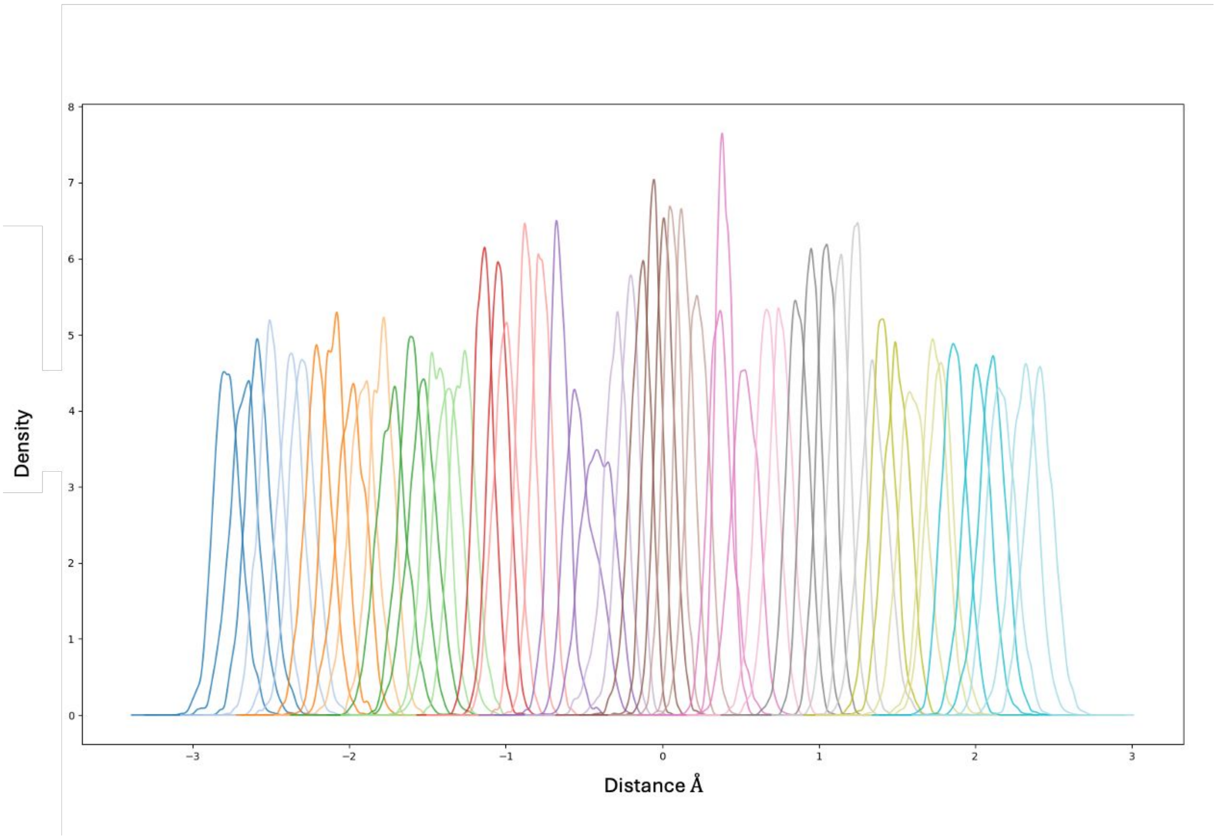

Figure S8: Density vs. umbrella sampling window for the reaction coordinate $d_{break2} - d_{water.}$

Table S4: The histogram distribution for the umbrella sampling windows of the deacylation stage.

RC_i / Å	min	Q1	median	Q3	max	mean	stdev	mean-ref	Q1-to-mean	Q3-to-mean
**-2.80**	-3.08	-2.84	-2.78	-2.72	-2.46	-2.78	0.09	0.02	0.06	0.06
**-2.70**	-2.99	-2.73	-2.67	-2.61	-2.35	-2.67	0.09	0.03	0.06	0.06
**-2.60**	-2.84	-2.64	-2.58	-2.53	-2.27	-2.58	0.09	0.02	0.06	0.05
**-2.50**	-2.79	-2.55	-2.50	-2.45	-2.21	-2.50	0.08	0.00	0.05	0.05
**-2.40**	-2.65	-2.44	-2.38	-2.32	-2.08	-2.38	0.08	0.02	0.06	0.06
**-2.30**	-2.58	-2.36	-2.30	-2.24	-2.02	-2.30	0.09	0.00	0.06	0.06
**-2.20**	-2.45	-2.25	-2.20	-2.14	-1.90	-2.20	0.08	0.00	0.06	0.06
**-2.10**	-2.40	-2.15	-2.10	-2.05	-1.84	-2.10	0.07	0.00	0.05	0.05
**-2.00**	-2.27	-2.05	-1.99	-1.92	-1.71	-1.99	0.09	0.01	0.06	0.07
**-1.90**	-2.21	-1.98	-1.92	-1.86	-1.67	-1.92	0.09	-0.02	0.06	0.06
**-1.80**	-2.03	-1.85	-1.79	-1.74	-1.52	-1.79	0.08	0.01	0.05	0.05
**-1.70**	-2.02	-1.79	-1.73	-1.67	-1.32	-1.73	0.10	-0.03	0.06	0.06
**-1.60**	-1.86	-1.66	-1.61	-1.55	-1.37	-1.61	0.08	-0.01	0.05	0.05
**-1.50**	-1.83	-1.60	-1.53	-1.47	-1.22	-1.53	0.09	-0.03	0.06	0.06
**-1.40**	-1.73	-1.50	-1.45	-1.39	-1.20	-1.45	0.08	-0.05	0.05	0.06
**-1.30**	-1.72	-1.43	-1.37	-1.31	-1.06	-1.37	0.09	-0.07	0.06	0.06
**-1.20**	-1.53	-1.33	-1.27	-1.22	-1.03	-1.27	0.08	-0.07	0.06	0.05
**-1.10**	-1.34	-1.19	-1.14	-1.10	-0.89	-1.14	0.06	-0.04	0.04	0.04
**-1.00**	-1.26	-1.09	-1.05	-1.00	-0.83	-1.05	0.07	-0.05	0.04	0.05

**-0.90**	-1.25	-1.05	-1.00	-0.95	-0.76	-1.00	0.08	-0.10	0.05	0.05
**-0.80**	-1.08	-0.91	-0.87	-0.83	-0.67	-0.87	0.06	-0.07	0.04	0.04
**-0.70**	-1.00	-0.81	-0.77	-0.73	-0.56	-0.77	0.06	-0.07	0.04	0.04
**-0.60**	-0.91	-0.70	-0.66	-0.62	-0.39	-0.66	0.07	-0.06	0.04	0.04
**-0.50**	-0.77	-0.59	-0.53	-0.45	-0.21	-0.52	0.10	-0.02	0.07	0.07
**-0.45**	-0.76	-0.48	-0.41	-0.34	-0.15	-0.41	0.10	0.04	0.07	0.07
**-0.40**	-0.61	-0.35	-0.30	-0.25	-0.09	-0.31	0.08	0.09	0.05	0.06
**-0.30**	-0.50	-0.26	-0.21	-0.17	0.01	-0.22	0.07	0.08	0.04	0.05
**-0.20**	-0.43	-0.19	-0.14	-0.09	0.05	-0.14	0.07	0.06	0.04	0.05
**-0.10**	-0.31	-0.10	-0.06	-0.02	0.12	-0.06	0.06	0.04	0.04	0.04
**0.00**	-0.21	-0.03	0.01	0.06	0.25	0.02	0.06	0.02	0.04	0.04
**0.10**	-0.14	0.02	0.06	0.10	0.27	0.06	0.06	-0.04	0.04	0.04
**0.20**	-0.05	0.10	0.14	0.18	0.32	0.14	0.06	-0.06	0.04	0.04
**0.30**	-0.02	0.18	0.23	0.28	0.47	0.23	0.07	-0.07	0.05	0.05
**0.40**	0.12	0.32	0.37	0.42	0.68	0.37	0.08	-0.03	0.05	0.05
**0.40**	0.23	0.36	0.39	0.43	0.57	0.39	0.05	-0.01	0.04	0.04
**0.50**	0.24	0.47	0.53	0.59	0.81	0.53	0.08	0.03	0.06	0.06
**0.60**	0.40	0.62	0.67	0.72	0.88	0.67	0.07	0.07	0.05	0.05
**0.70**	0.50	0.70	0.75	0.81	1.02	0.75	0.08	0.05	0.05	0.05
**0.80**	0.62	0.82	0.86	0.91	1.09	0.86	0.07	0.06	0.05	0.05
**0.90**	0.76	0.91	0.95	1.00	1.20	0.95	0.06	0.05	0.04	0.04
**1.00**	0.86	1.20	1.05	1.09	1.25	1.05	0.06	0.05	0.04	0.04
**1.10**	0.92	1.09	1.13	1.18	1.36	1.13	0.07	0.03	0.04	0.05
**1.20**	1.05	1.19	1.23	1.27	1.45	1.23	0.06	0.03	0.04	0.04
**1.30**	1.08	1.29	1.35	1.41	1.67	1.35	0.09	0.05	0.06	0.06
**1.40**	1.15	1.36	1.41	1.46	1.65	1.41	0.08	0.01	0.05	0.05
**1.50**	1.22	1.44	1.50	1.56	1.76	1.50	0.08	0.00	0.06	0.06
**1.60**	1.31	1.54	1.60	1.66	1.95	1.60	0.09	0.00	0.06	0.06
**1.70**	1.45	1.68	1.74	1.79	2.09	1.74	0.08	0.04	0.05	0.05
**1.80**	1.50	1.72	1.78	1.83	2.04	1.78	0.09	-0.02	0.06	0.06
**1.90**	1.61	1.81	1.87	1.92	2.14	1.87	0.08	-0.03	0.05	0.05
**2.00**	1.71	1.95	2.01	2.07	2.35	2.01	0.09	0.01	0.06	0.06
**2.10**	1.83	2.05	2.11	2.16	2.44	2.11	0.09	0.01	0.06	0.06
**2.20**	1.89	2.10	2.16	2.23	2.47	2.17	0.09	-0.03	0.06	0.06
**2.30**	2.03	2.26	2.32	2.37	2.64	2.31	0.09	0.01	0.06	0.06
**2.40**	2.13	2.35	2.41	2.47	2.72	2.41	0.09	0.01	0.06	0.06

Table S5: Details for the umbrella sampling simulations carried out for the acylation and deacylation steps : the center of application of the harmonic bias, RC_i, and the corresponding harmonic spring constant, K_i, for an harmonic potential of form $U_i(RC) = K_i(RC - RC_i)^2$.

$\boldsymbol{RC_{ACYLATION} = d_{break1} - d_{nuc}}$	$\boldsymbol{RC_{DEACYLATION} = d_{break2} - d_{water}}$

$\boldsymbol{RC_i}$ / Å	$\frac{K_i}{kcal \cdot mol^{-1} \cdot Å^{-2}}$	RC_i / Å	$\frac{K_i}{kcal \cdot mol^{-1} \cdot Å^{-2}}$
-2.20	50.0	-2.80	50.0
-2.10	50.0	-2.70	50.0
-2.00	50.0	-2.60	50.0
-1.90	50.0	-2.50	50.0
-1.80	50.0	-2.40	50.0
-1.70	50.0	-2.30	50.0
-1.60	50.0	-2.20	50.0
-1.50	50.0	-2.10	50.0
-1.40	50.0	-2.00	50.0
-1.30	50.0	-1.90	50.0
-1.20	50.0	-1.80	50.0
-1.10	50.0	-1.70	50.0
-1.00	100.0	-1.60	50.0
-0.90	100.0	-1.50	50.0
-0.80	100.0	-1.40	50.0
-0.70	100.0	-1.30	50.0
-0.60	100.0	-1.20	50.0
-0.50	100.0	-1.10	100.0
-0.40	100.0	-1.00	100.0
-0.30	100.0	-0.90	50.0
-0.20	50.0	-0.80	100.0
-0.10	50.0	-0.70	100.0
0.00	50.0	-0.60	100.0
0.10	50.0	-0.50	100.0
0.20	50.0	-0.45	100.0
0.30	100.0	-0.40	100.0
0.40	100.0	-0.30	100.0
0.50	100.0	-0.20	100.0
0.60	100.0	-0.10	50.0
0.70	100.0	0.00	50.0
0.80	100.0	0.10	50.0
0.90	50.0	0.20	100.0
1.00	50.0	0.30	100.0
1.10	50.0	0.40	200.0
1.20	50.0	0.40	100.0
1.30	50.0	0.50	100.0
1.40	50.0	0.60	100.0
1.50	50.0	0.70	100.0
1.60	50.0	0.80	100.0
1.70	50.0	0.90	100.0
1.80	50.0	1.00	100.0
1.90	50.0	1.10	100.0

2.00	50.0	1.20	100.0
2.10	50.0	1.30	50.0
2.20	50.0	1.40	50.0
2.30	50.0	1.50	50.0
2.40	50.0	1.60	50.0
2.50	50.0	1.70	50.0
		1.80	50.0
		1.90	50.0
		2.00	50.0
		2.10	50.0
		2.20	50.0
		2.30	50.0
		2.40	50.0

Table S6: Average free energies with standard deviations across cumulative forward timeseries (given in kcal·mol-^{1}) obtained across reaction states (R → TS1 → TI1 → TS2 → AI) for the acylation stage. Grey shading indicates the standard deviation from cumulative forward time analysis, emphasizing the stability of energies near 1 kcal·mol-^{1}. Red colored cells correspond to the simulation time per window considered for equilibration.

Time	R	TS1	TI1	TS2	AI
**0ps to 2ps**	0.00	5.98	4.16	7.61	1.69
**0ps to 4ps**	0.00	6.8	4.61	8.13	1.72
**0ps to 6ps**	0.00	6.66	4.53	7.93	0.00
**0ps to 8ps**	0.00	6.99	4.82	7.79	-0.18
**0ps to 10ps**	0.00	7.05	4.83	7.49	-1.25
**0ps to 12ps**	0.00	7.22	5.06	7.68	-1.52
**0ps to 14ps**	0.00	7.35	5.24	7.88	-1.42
**0ps to 16ps**	0.00	7.35	5.21	7.63	-2.00
**0ps to 18ps**	0.00	7.42	5.19	7.48	-2.47
**0ps to 20ps**	0.00	7.46	5.25	7.52	-2.56
**STDV**		**0.46**	**0.37**	**0.21**	**0.95**

Table S7: Average free energies with standard deviations across cumulative reverse timeseries (given in kcal·mol-^{1}) obtained across reaction states (R → TS1 → TI1 → TS2 → AI) for the acylation stage. Grey shading indicates the standard deviation from cumulative reverse time analysis, emphasizing the stability of energies near 1 kcal·mol-^{1}.

Time	RC	TS1	TI1	TS2	AI
**0ps to 20ps**	0.00	7.46	5.25	7.52	-2.56
**2ps to 20ps**	0.00	7.63	5.40	7.57	-3.05
**4ps to 20ps**	0.00	7.63	5.40	7.48	-3.65
**6ps to 20ps**	0.00	7.78	5.51	7.48	-3.99
**8ps to 20ps**	0.00	7.74	5.48	7.41	-4.21
**10ps to 20ps**	0.00	7.87	5.65	7.64	-3.85
**12ps to 20ps**	0.00	7.82	5.52	7.37	-4.09

**14ps to 20ps**	0.00	7.74	5.25	6.83	-5.16
**16ps to 20ps**	0.00	7.97	5.47	7.23	-4.71
**18ps to 20ps**	0.00	7.98	5.87	8.03	-3.39
**STDV**		**0.16**	**0.18**	**0.30**	**0.76**

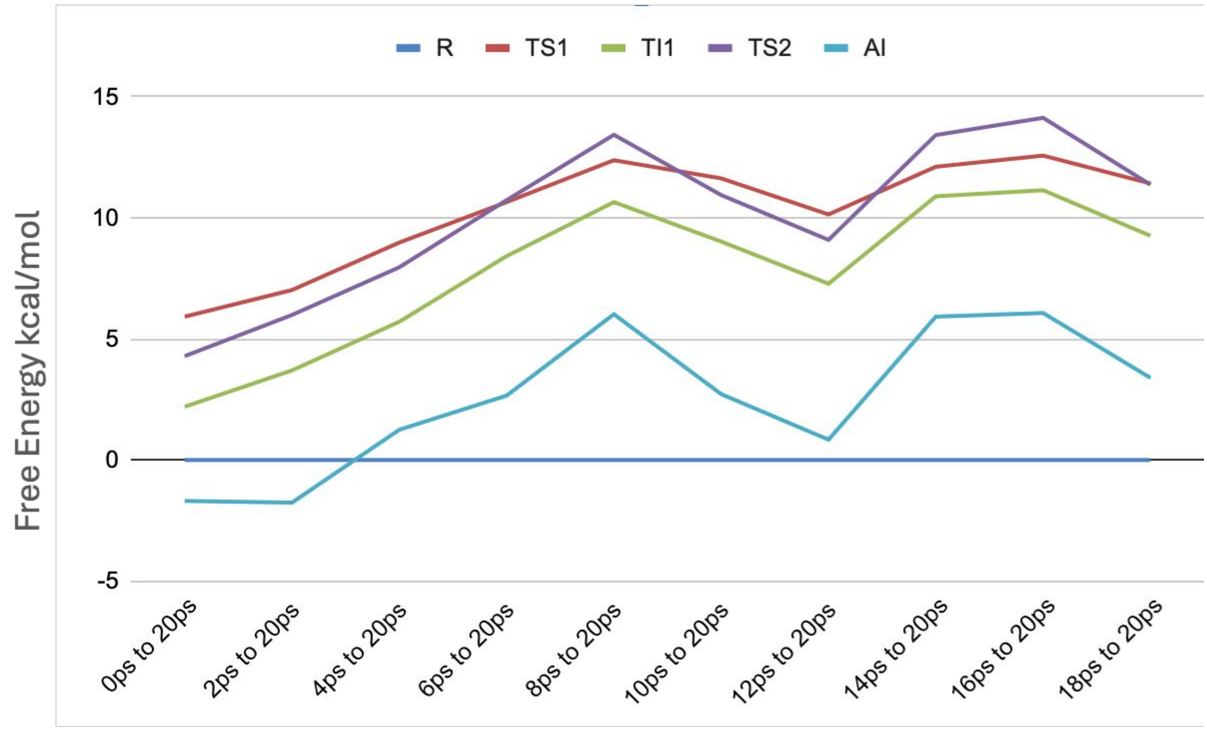

Figure S9: Free energies by block analysis (given in kcal·mol-¹) obtained across reaction states (R → TS1 → TI1 → TS2 → AI) for the acylation stage. We observe that the energies oscillate in a regular, repeating way across the defined blocks.

Table S8: Average free energies with standard deviations across cumulative forward timeseries (given in kcal·mol-¹) obtained across reaction states (AI → TS3 → TI2 → TS4 → PC) for the deacylation stage. Grey shading indicates the standard deviation from block-wise time analysis, emphasizing the stability of energies near 1 kcal·mol-¹. Red colored cells correspond to the simulation time per window considered for equilibration.

Time	AI	TS3	TI2	TS4	PC
**0ps to 2ps**	0.00	14.70	12.34	19.82	10.93
**0ps to 4ps**	0.00	13.84	11.85	18.73	9.67
**0ps to 6ps**	0.00	13.36	11.14	17.05	8.42
**0ps to 8ps**	0.00	12.51	9.57	15.10	6.42
**0ps to 10ps**	0.00	11.97	8.46	13.76	5.07
**0ps to 12ps**	0.00	11.72	8.03	13.00	4.46
**0ps to 14ps**	0.00	11.45	7.45	12.18	3.6
**0ps to 16ps**	0.00	11.37	7.14	11.71	3.12
**0ps to 18ps**	0.00	11.29	6.85	11.34	2.83
**0ps to 20ps**	0.00	11.26	6.54	10.96	2.50
**STDV**		**0.74**	**1.05**	**1.06**	**0.99**

Table S9: Average free energies with standard deviations across cumulative reverse timeseries (given in kcal·mol-¹) obtained across reaction states (AI → TS3 → TI2 → TS4 → PC) for the deacylation stage. Grey shading indicates the standard deviation from cumulative reverse time analysis, emphasizing the stability of energies near 1 kcal·mol-¹.

Time	AI	TS3	TI2	TS4	PC
**0ps to 20ps**	0.00	11.26	6.54	10.96	2.50
**2ps to 20ps**	0.00	10.94	5.88	10.04	1.52
**4ps to 20ps**	0.00	10.72	5.24	9.15	0.63
**6ps to 20ps**	0.00	10.53	4.68	8.50	-0.05
**8ps to 20ps**	0.00	10.58	4.58	8.34	-0.22
**10ps to 20ps**	0.00	10.62	4.64	8.33	-0.24
**12ps to 20ps**	0.00	10.68	4.44	8.20	-0.51
**14ps to 20ps**	0.00	11.06	4.72	8.54	-0.02
**16ps to 20ps**	0.00	11.01	4.35	8.21	0.02
**18ps to 20ps**	0.00	11.30	4.07	7.95	-0.28
**STDV**		**0.28**	**0.76**	**0.96**	**0.96**

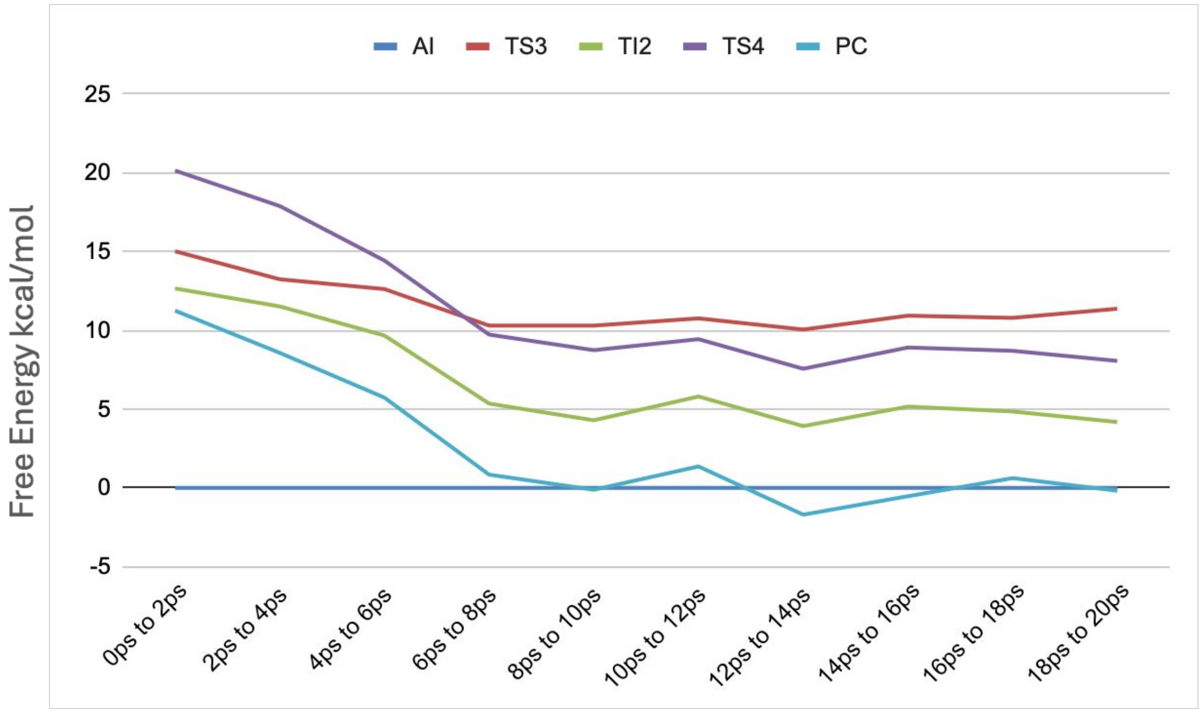

Figure S10: Free energies by block analysis (given in kcal·mol-¹) obtained across reaction states (AI → TS3 → TI2 → TS4 → PC) for the deacylation stage. We observe that the energies oscillate in a regular, repeating way across the defined blocks.

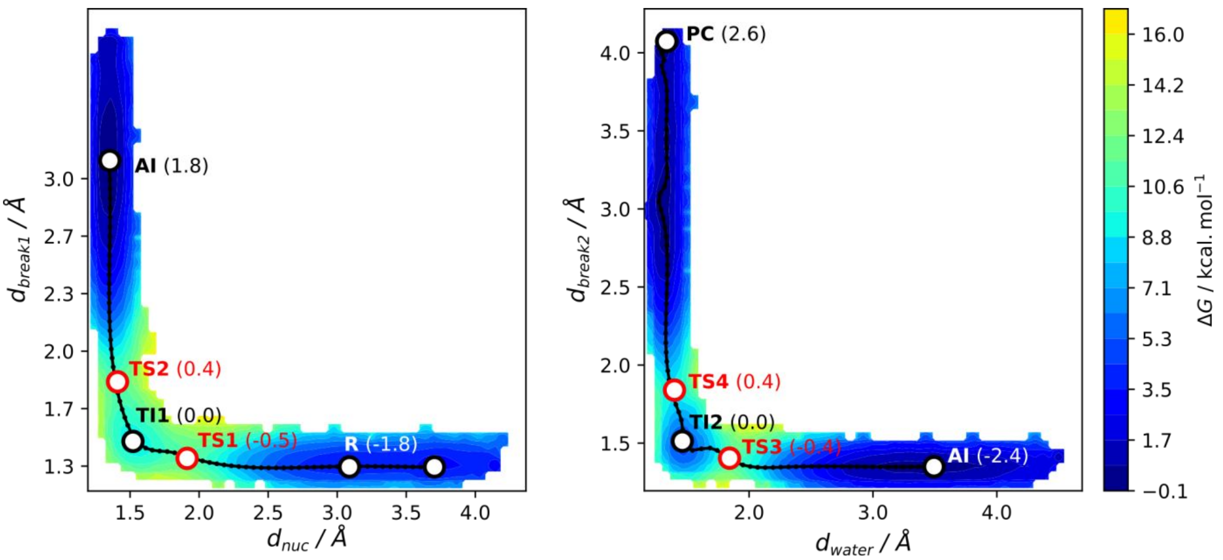

Figure S11: 2D free energy projection considering the distances to define the reaction coordinate for the acylation step (RC_acylation, on the left) and the deacylation step (RC_deacylation, on the right). White dots correspond to stationary points identified throughout the reaction, and the black line connecting them corresponds to the corresponding interpolated minimum free energy path. Values in parentheses correspond to the value of the reaction coordinate at each stationary point.

Table S10: Average distances (given in Å) obtained across reaction states (R → TS1 → TI1 → TS2 → AI) for the acylation stage.

Distances	R	TS1	TI1	TS2	AI
**S171Oγ-CPET**	3.06 ± 0.05	1.84 ± 0.05	1.51 ± 0.03	1.43 ± 0.04	1.36 ± 0.03
**OXPET-F98NH**	2.05 ± 0.23	1.83 ± 0.19	1.71 ± 0.16	1.86 ± 0.16	2.05 ± 0.19
**OXPET-M172NH**	2.89 ± 0.31	2.14 ± 0.21	2.01 ± 0.20	2.20 ± 0.23	2.06 ± 0.20
**CPET-OCPET**	1.36 ± 0.03	1.42 ± 0.04	1.46 ± 0.03	1.79 ± 0.05	3.01 ± 0.02
**S171Oγ-Hγ**	1.03 ± 0.04	1.52 ± 0.18	1.86 ± 0.23	2.53 ± 0.20	2.94 ± 0.20
**H249Nϵ-S171Hγ**	1.71 ± 0.17	1.13 ± 0.10	1.06 ± 0.04	1.14 ± 0.12	1.83 ± 0.18
**OCPET-S171Hγ**	3.45 ± 0.31	2.82 ± 0.25	2.59 ± 0.37	1.53 ± 0.22	1.02 ± 0.04
**H249Hδ-D217O**	1.58 ± 0.14	1.30 ± 0.18	1.18 ± 0.12	1.35 ± 0.20	1.63 ± 0.16

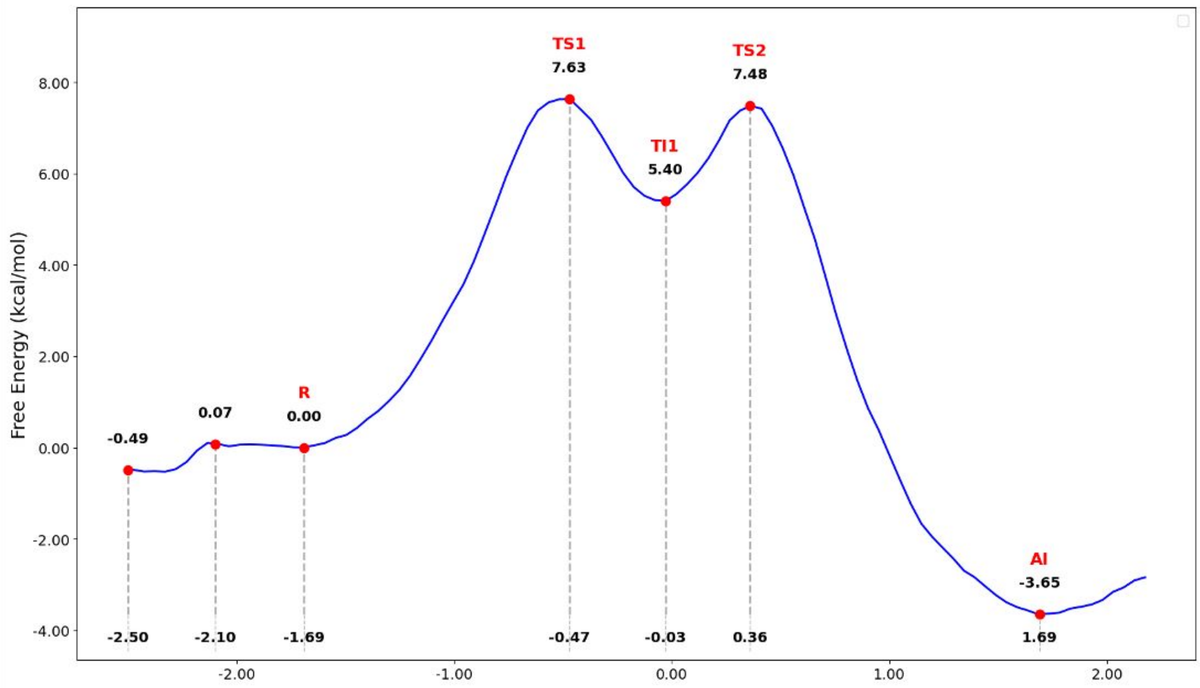

Figure S12: The corresponding free energy profile for the acylation stage displays free energy barriers and the reaction free energy with corresponding reaction coordinates (grey dotted line). Points where the curve presents a transient increase in free energy of approximately ≈ 0.56 kcal·mol-[1] in the pre-reactant stage are highlighted in red.

Table S11: Average distances (given in Å) obtained for the pre-reactive stage for the acylation. The average distances, before and after the transient increase in free energy, suggest a brief misorientation of the S171-hydroxyl, which initially approached the nearby ester-oxo group of the PET dimer instead of the intended the Nε atom of H249. This unfavorable interaction introduced a minor energetic penalty, which was rapidly resolved by the reorientation of the S171-hydroxyl correctly toward the Nε atom of H249, allowing the reaction to proceed along the reaction pathway.

	$d_{BREAK} - d_{NUC}$ / Å	
**Distances**	**-2.50**	**-2.10**
**S171Oγ-CPET**	3.86±0.06	3.47±0.06
**H249Nϵ-S171Hγ**	3.10±0.55	1.78±0.21
**OXPET- S171Hγ**	2.24±0.60	3.34±0.31
**OXPET-F98NH**	3.19±0.65	2.34±0.38
**OXPET-M172NH**	3.92±0.39	3.27±0.20

Table S12: Average distances (given in Å) obtained across reaction states (AI → TS3 → TI2 → TS4 → PC) for the deacylation stage.

Distances	AI	TS3	TI2	TS4	PC
**O_{wat}-C_{PET}**	3.43 ± 0.05	1.89 ± 0.05	1.47 ± 0.04	1.40 ± 0.03	1.32 ± 0.03
**OXPET-F98NH**	1.98 ± 0.20	1.83 ± 0.15	1.64 ± 0.14	1.72 ± 0.15	1.91 ± 0.18
**OXPET-M172NH**	2.12 ± 0.24	2.01 ± 0.19	1.85 ± 0.18	1.94 ± 0.20	2.39 ± 0.30
**C_{PET}- S171Oγ**	1.36 ± 0.03	1.42 ± 0.04	1.49 ± 0.04	1.83 ± 0.05	3.08 ± 0.05
**O_{wat}- H_{wat}**	1.02 ± 0.04	1.18 ± 0.21	2.02 ± 0.48	2.82 ± 0.17	3.20 ± 0.20
**H249Nϵ- H_{wat}**	1.86 ± 0.32	1.49 ± 0.24	1.06 ± 0.04	1.17 ± 0.14	2.11 ± 0.18

**H_{wat}-S171Oγ**	3.22 ± 0.30	2.62 ± 0.17	2.42 ± 0.38	1.51 ± 0.26	1.02 ± 0.04
**H249Hδ-D217O**	1.67 ± 0.15	1.56 ± 0.23	1.23 ± 0.18	1.35 ± 0.20	1.52 ± 0.21

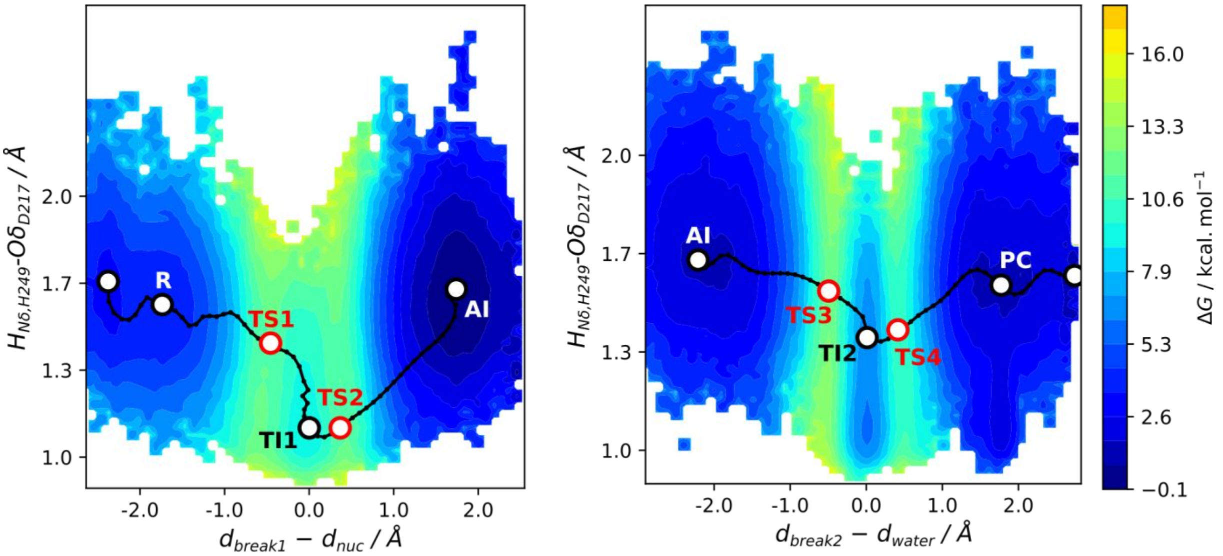

Figure S13: 2D free energy projection of the distance describing proton transfer events between H249 and D217 against the reaction coordinate for the acylation step (RC_acylation, on the left) and the deacylation step (RC_deacylation, on the right). White dots correspond to stationary points identified throughout the reaction, and the black line connecting them corresponds to the corresponding interpolated minimum free energy path.

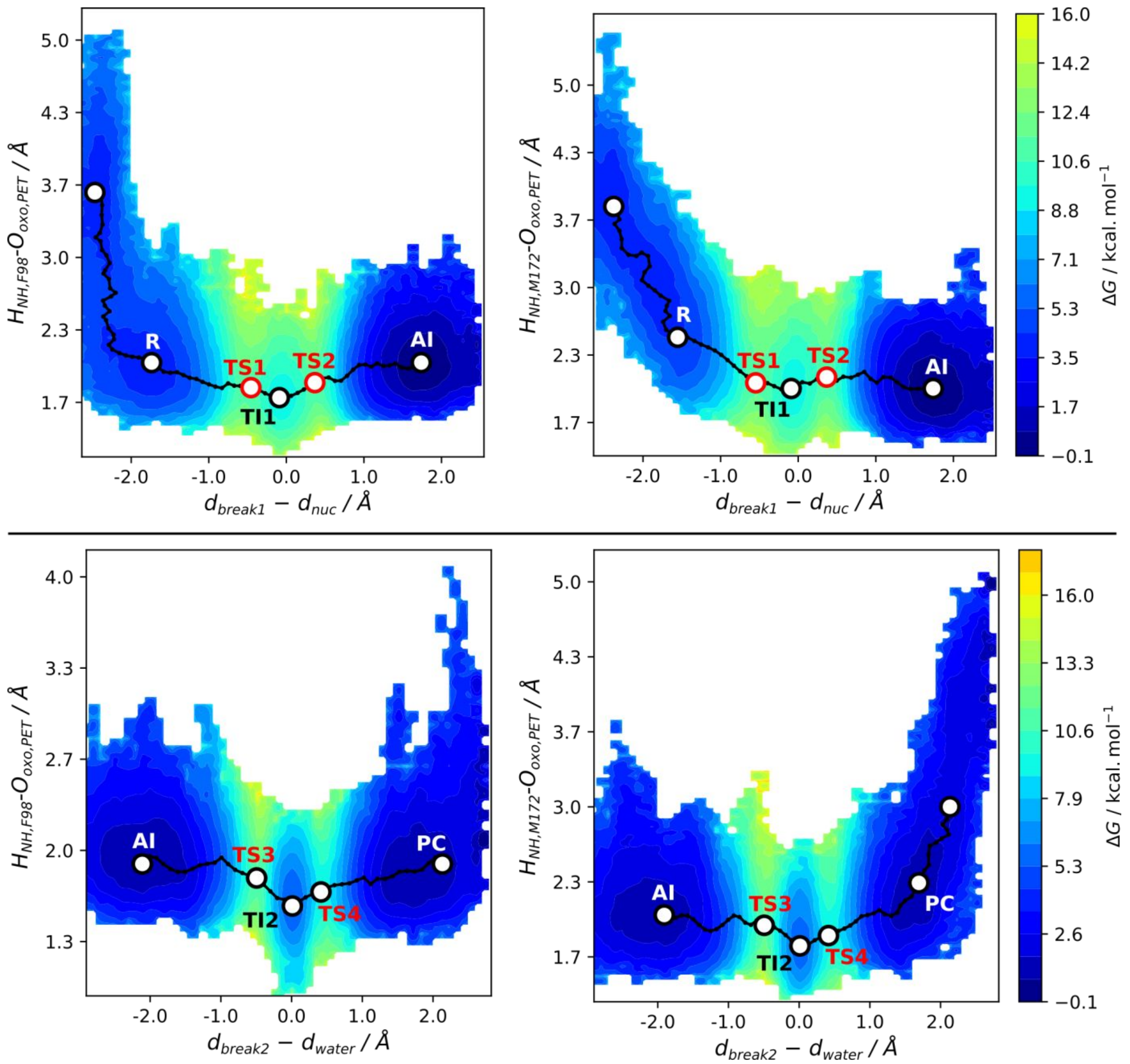

Figure S14: 2D free energy projection for the change of the oxyanion hole distances involving F98 and M172, along the reaction coordinate used for the acylation step (RC_acylation, on the top) and for the deacylation step (RC_deacylation, on the bottom). White dots correspond to stationary points identified throughout the reaction, and the black line connecting them corresponds to the corresponding interpolated minimum free energy path.

REFERENCES

1. Zeng W, Li X, Yang Y, et al. Substrate-Binding Mode of a Thermophilic PET Hydrolase and Engineering the Enzyme to Enhance the Hydrolytic Efficacy. ACS Catal. 2022;12(5):3033-3040. doi:10.1021/acscatal.1c05800
